# Genomic Regions Involved in Differences in Eating and Cooking Quality Other than *Wx* and *Alk* Genes between *indica* and *japonica* Rice Cultivars

**DOI:** 10.1186/s12284-020-00447-8

**Published:** 2021-01-07

**Authors:** Kiyosumi Hori, Keitaro Suzuki, Haruka Ishikawa, Yasunori Nonoue, Kazufumi Nagata, Shuichi Fukuoka, Junichi Tanaka

**Affiliations:** 1 Institute of Crop Science, NARO, 2-1-2 Kannondai, Tsukuba, Ibaraki, 305-8518 Japan; 2 College of Agriculture, Ibaraki University, 3-21-1 Chuo, Ami, Ibaraki, 300-0393 Japan; 3 Present address: St. Marianna University School of Medicine, 2-16-1 Sugao, Miyamae, Kawasaki, Kanagawa 216-8511 Japan; 4 Graduate School of Life and Environmental Science, University of Tsukuba, 2-1-2 Kannondai, Tsukuba, Ibaraki, 305-8518 Japan

**Keywords:** Rice, Eating quality, Quantitative trait loci, Chromosome segment substitution lines

## Abstract

**Background:**

In temperate rice cultivation regions, *japonica* rice cultivars are grown preferentially because consumers deem them to have good eating quality, whereas *indica* rice cultivars have high grain yields and strong heat tolerance but are considered to have poor eating quality. To mitigate the effects of global warming on rice production, it is important to develop novel rice cultivars with both desirable eating quality and resilience to high temperatures. Eating quality and agronomic traits were evaluated in a reciprocal set of chromosome segment substitution lines derived from crosses between a *japonica* rice cultivar ‘Koshihikari’ and an *indica* rice cultivar ‘Takanari’.

**Results:**

We detected 112 QTLs for amylose and protein contents, whiteness, stickiness, hardness and eating quality of cooked rice grains. Almost of ‘Koshihikari’ chromosome segments consistently improved eating quality. Among detected QTLs, six QTLs on chromosomes 1–5 and 11 were detected that increased whiteness and stickiness of cooked grains or decreased their hardness for 3 years. The QTLs on chromosomes 2–4 were not associated with differences in amylose or protein contents. QTLs on chromosomes 1–5 did not coincide with QTLs for agronomic traits such as heading date, culm length, panicle length, spikelet fertility and grain yield. Genetic effects of the detected QTLs were confirmed in substitution lines carrying chromosome segments from five other *indica* cultivars in the ‘Koshihikari’ genetic background.

**Conclusion:**

The detected QTLs were associated with differences in eating quality between *indica* and *japonica* rice cultivars. These QTLs appear to be widely distributed among *indica* cultivars and to be novel genetic factors for eating quality traits because their chromosome regions differed from those of the *GBSSI* (*Wx*) and *SSIIa* (*Alk*) genes. The detected QTLs would be very useful for improvement of eating quality of *indica* rice cultivars in breeding programs.

**Supplementary Information:**

The online version contains supplementary material available at 10.1186/s12284-020-00447-8.

## Background

Rice (*Oryza sativa* L.) is a staple food for nearly half of the world population (GriSP (Global Rice Science Partnership), 2013). This species is divided into two main subspecies, *indica* and *japonica*, which differ in their morphological and physiological characteristics (Khush 1997). *Indica* is grown mainly in tropical regions, whereas *japonica* is grown in temperate regions such as China, Korea and Japan. The two subspecies have been suggested to have different domestication origins because, for example, seed shattering is reduced by the loss of function of different genes (Konishi et al. 2006; Li et al. 2006) and there are multiple sequence polymorphisms throughout their genomes (Garris et al. 2005; Wang et al. 2018), indicating considerable intraspecific differentiation. Generally, F_1_ plants from crosses between the two subspecies show low fertility, and segregating populations such as F_2_ often show sterility and hybrid weakness (Matsubara et al. 2007; Yamamoto et al. 2007). Therefore, in modern rice breeding, programs for *indica*-background breeding and for *japonica*-background breeding are separate, although breeders use common agronomically important genes for disease resistance, plant height and stress tolerance in these programs.

The International Rice Research Institute (IRRI) developed an *indica* cultivar ‘IR8’ by introducing the semi-dwarf gene *sd1* derived from ‘Dee-geo-woo-gen’; ‘IR8’ has played the main role in the Green Revolution in rice (Khush 1999). Since then, IRRI has developed many high-yielding *indica* cultivars (e.g., ‘IR64’), which are widely cultivated, mainly in tropical regions (Mackill and Khush 2018). Japanese *indica* cultivars such as ‘Takanari’ and ‘Hokuriku 193’ are derived from crosses with the IRRI cultivars and have significantly higher yields than Japanese *japonica* cultivars such as ‘Koshihikari’, even in temperate climates (Imbe et al. 2004; Goto et al. 2009). Global warming is likely to reach 1.5 °C between 2030 and 2052 if it continues at the current rate (IPCC (Intergovernmental Panel on Climate Change), 2018). In general, *indica* cultivars are adapted to higher temperatures in low latitudes better than *japonica* cultivars. Therefore, the genetic backgrounds of *indica* rice will be more suitable for cultivation in temperate regions, which are likely to experience an increase in temperature during rice growth seasons in the future (IPCC (Intergovernmental Panel on Climate Change), 2018).

*Indica* rice cultivars are preferred by consumers in most rice cultivation areas, but not in Northeast Asia, where people are accustomed to eating *japonica* rice (Juliano et al. 1993; Calingacion et al. 2014). Many consumers in such countries as China, Korea and Japan tend to prefer the softness and strong stickiness of cooked grains of *japonica* rice cultivars. Almost all *indica* cultivars have low stickiness and high hardness of cooked grains (Calingacion et al. 2014; Hori and Yano 2013). One of main genetic factors controlling stickiness and hardness of cooked rice grains are allelic differences in the *Waxy* (*Wx*, *GBSSI*) gene involved in amylose synthesis in rice endosperm (Tan et al. 1999; Wan et al. 2004; Tian et al. 2005; Takeuchi et al. 2007; Su et al. 2011; Yang et al. 2018; Park et al. 2019; Yang et al. 2020). In general, *indica* cultivars have the *Wx*^*a*^ allele, which results in high amylose content, whereas *japonica* cultivars have the *Wx*^*b*^ allele, which results in moderate amylose content (Juliano et al. 1993). Several other *Wx* alleles have also been reported in *indica* cultivars such as *Wx*^*lv*^ and *Wx*^*in*^ with high amylose content, and in *japonica* cultivars such as *Wx*^*la*^ and *Wx*^*mq*^ with low amylose content (Sato et al. 2002; Zhou et al. 2015; Zhang et al. 2019; Zhou et al. 2020). Chromosome region at the *Wx* gene showed high recombination rate and various gene alleles were generated by intragenic recombination. The *Alkali degradation* (*Alk*, *SSIIa*) gene involved in amylopectin chain elongation is also controlling eating quality of cooked rice grains by altering starch characteristics such as amylose content, gelatinization temperature and gel consistency (Umemoto et al. 2002; Umemoto 2018). Haplotype analysis revealed that these phenotypic differences were significantly correlated with allelic differences of the *Alk* gene between *indica* and *japonica* rice cultivars (Umemoto et al. 2004). In general, *indica* cultivars have the *Alk* allele, which is strong functional allele and results in high gelatinization temperature, whereas *japonica* cultivars have the *alk* allele, which is weak functional allele and results in low gelatinization temperature. Another *Alk* allele of *Alk*^*b*^ is reported as weak functional allele with low gelatinization temperature and gel consistency both in *indica* and *japonica* cultivars (Chen et al. 2020). Allelic difference of the *Alk* gene largely changed on eating and cooking qualities in near-isogenic lines (NILs) introducing the *Wx*^*b*^ and *wx* gene alleles as compared with in NILs introducing the *Wx*^*a*^ gene allele (Umemoto 2018).

Amylose and protein contents of a Japanese *indica* cultivar ‘Takanari’ are not much different from those of the *japonica* cultivar ‘Koshihikari’, which is a leading cultivar in Japan (Hori et al. 2016; Iijima et al. 2019). However, ‘Takanari’ has the *Wx*^*b*^ allele introduced from *japonica* cultivars (Aoki et al. 2015). ‘Takanari’ has harder cooked grains and significantly inferior taste in comparison with typical Japanese *japonica* rice cultivars including ‘Koshihikari’. Therefore, the difference in eating quality between *indica* and *japonica* rice cultivars cannot be explained only by amylose and protein contents, but other major genetic factors related to eating quality are hardly known. To improve eating quality of *indica* rice cultivars, it is necessary to detect novel genetic factors associated with eating quality.

*indica* rice cultivars are considered unsuitable for consumers in Northeast Asia because of their eating and cooking characteristics. Development of novel rice cultivars with the *indica* genetic background, good eating quality and high yield in Northeast Asia would be an effective solution to the possible food-supply crisis caused by global warming in the future. In this study, we attempted to detect QTLs for eating quality in chromosome segment substitution lines (CSSLs) derived from crosses between ‘Koshihikari’ and ‘Takanari’ (Takai et al. 2014). We found multiple QTLs related to differences in eating quality between the two cultivars, and some of these QTLs were not associated with amylose or protein contents. These CSSLs can be promising materials to introduce novel genetic factors for eating quality into *indica* rice cultivars.

## Materials and Methods

### Plant Materials

To detect QTLs involved in the control of eating-quality traits, we used a reciprocal set of CSSLs derived from crosses between a *japonica* rice cultivar ‘Koshihikari’ and an *indica* rice cultivar ‘Takanari’ (Takai et al. 2014). Forty-one CSSLs covered most of the ‘Takanari’ genome in the ‘Koshihikari’ genetic background and 39 CSSLs covered the ‘Koshihikari’ genome in the ‘Takanari’ genetic background. Genotype information of individual CSSLs is available in Takai et al. (2014).

Eight CSSLs derived from crosses between ‘Koshihikari’ as a recurrent parent and five *indica* rice cultivars—‘Naba’ (WRC5), ‘Bleiyo’ (WRC63), ‘Bei Khe’ (WRC3), ‘Tupa 121–3’ (WRC32) and ‘Basilanon’ (WRC44) (Kojima et al. 2005)—were selected to investigate whether the detected QTLs for eating quality traits were common among *indica* rice cultivars. In these CSSLs of the ‘Koshihikari’ genetic background, chromosome segments derived from these *indica* rice cultivars cover the regions of detected QTLs. Genotype information of the eight CSSLs were indicated in Supplementary Table S1.

To reveal the genotypes of the QTL (*qWH1*) region that enhances the whiteness of cooked rice grains, which is adjacent to the *sd1* gene, we selected five leading cultivars in Japan: ‘Koshihikari’, ‘Hitomebore’, ‘Hinohikari’, ‘Akitakomachi’ and ‘Nanatsuboshi’ (Kobayashi et al. 2018), and six high-yielding and good-eating-quality cultivars that were released recently (2011–2019) in Japan: ‘Akidawara’, ‘Hoshijirushi’, ‘Mizuhonokagayaki’, ‘Tsukiakari’, ‘Natsuhonoka’ and ‘Nijinokirameki’.

### Evaluation of Eating Quality Traits

All plants of CSSLs and parental cultivars ‘Koshihikari’ and ‘Takanari’ were grown in an experimental field at the Institute of Crop Science, NARO, Tsukubamirai, Japan (36.01°N, 140.02°E). CSSLs of the ‘Koshihikari’ genetic background were grown in 2016 and 2017, and CSSLs of the ‘Takanari’ genetic background in 2018. Japanese leading cultivars and recently developed cultivars were grown in 2018. One-month-old seedlings of all CSSLs and cultivars were transplanted in mid-May at one plant per hill in plots with a double row for each line; there was 15 cm between plants and 30 cm between rows. Cultivation management followed the standard procedures used at the institute.

Eating quality traits were evaluated by instrumental methods according to Hori et al. (2016) and Iijima et al. (2019). Apparent amylose content was determined by using an Auto Analyzer II (Bran+Luebbe Co. Ltd., Norderstedt, Germany). Crude protein content was determined by the combustion method with an induction furnace at 900 °C (American Association of Cereal Chemists International, Approved Method 46–30.01). Whiteness and grain qualities were evaluated with a Rice Grain Analyzer RGQI20B (Satake Co., Ltd., Hiroshima, Japan). Eating quality score was measured in a Cooked Rice Taste Analyzer STA1A (Satake Co., Ltd.). This instrument has been used to estimate eating quality scores by measuring transmitted light volume and reflection light volume of cooked rice grains under three wavelengths (Mikami 2009). Physical properties of cooked grains were measured by the high-compression/low-compression method with a Tensipresser MyBoy texture analyzer (Takemoto Electric Co., Tokyo, Japan). These instrumental methods showed significant correlations with the eating quality scores by the sensory tests (Okadome 2005; Mikami 2009; Kwon et al. 2011; Hori et al. 2016).

### Scoring of Agronomic Traits

For each CSSL and parental cultivar, days to heading was defined as the number of days from sowing to heading of half of the plants. Culm length, panicle length, panicle number, spikelet fertility (ratio of the number of sterile and fertile grains per panicle) and unhulled grain weight per plant were measured for five plants per CSSL and parental cultivar at maturity stage.

### Statistical and Genetic Analyses

Eating quality traits and agronomic traits of CSSLs were compared with those of each recurrent parent, ‘Koshihikari’ or ‘Takanari’, by using the Dunnett’s multiple comparison procedure provided by the JMP 11 software (SAS Institute Inc., NC, USA). In the Dunnett’s tests, ‘Koshihikari’ was used as a control for 41 CSSLs of ‘Takanari’ and 8 CSSLs of other *indica* cultivars in the ‘Koshihikari’ genetic background, and ‘Takanari’ was used as a control for 39 CSSLs in the ‘Takanari’ genetic background. QTLs were declared present when individual trait scores were significantly different between the line and the recurrent parent.

### DNA Marker Genotyping

Total genomic DNA of Japanese leading rice cultivars and recently developed rice cultivars was extracted from leaves using the CTAB method (Hori et al. 2012) and a DNA sui-sui S kit (Rizo Inc., Tsukuba, Ibaraki, Japan). The DNA markers described in Bao et al. (2006) and Hiratsuka et al. (2010) were used for determining the *Alk* gene allele in ‘Koshihikari’ and ‘Takanari’. We selected eight DNA markers—simple sequence repeats, insertion/deletions (InDels) and the *sd1* gene—that were polymorphic between ‘Koshihikari’ and ‘Takanari’. Simple sequence repeat markers of RM11716, RM11837, RM12168 and RM12263 were selected from IRGSP (International Rice Genome Sequencing Project) (2005). Oligoribonucleotide sequences of InDel markers and the *sd1* gene marker were 5′-GTGATCAATGTCGAGATAACGTTCC-3′ and 5′-GGTAAAAGGATTAGAGCACCGCTAC-3′ (JI_indel_01), 5′-TTTCAGGTAGGCATCACCAATAGAG-3′ and 5′-CTCCGTCCGAGGTGTCATAAATTAG-3′ (JI_indel_02), 5′-ATGCCGTTAATAGAATGGGAATACG-3′ and 5′-AGATCAAATCGTCAATGTGGAACAC-3′ (JI_indel_03), and 5′-ACGCACGGGTTCTTCCAGGTGT-3′ and 5′-GAGCGGGAGGCGGAAGAAGTC-3′ (*sd1*).

## Results

### QTLs for Eating Quality Traits in CSSLs of the ‘Koshihikari’ Genetic Background

The phenotypes of the CSSLs of the ‘Koshihikari’ genetic background varied widely in eating quality traits (amylose and protein contents, eating quality score, stickiness and hardness of cooked rice grains, and grain whiteness; Table 1, Supplementary Tables S2 and S3, Supplementary Figure 1). In comparison with ‘Koshihikari’, 44 QTLs in 2016 and 32 QTLs in 2017 were associated with significant differences in all six eating quality traits analyzed (Table 2). Genomic regions of almost all QTLs were consistent between 2016 and 2017.

**Table 1:** Eating-quality traits on chromosome segment substitution lines (CSSLs) in the ‘Koshihikari’ genetic background in 2016 and 2017

		2016						2017				
				Cooked Rice Taste Analyzer	Tensipresser		Rice Grain Image Analyzer		Cooked Rice Taste Analyzer	Tensipresser		Rice Grain Image Analyzer
		Amylose content	Protein content	Eating quality score	Stickiness S1	Hardness H2	Whiteness	Amylose content	Eating quality score	Stickiness S1	Hardness H2	Whiteness
Cultivar and Line No.	Donor segment	(%)	(%)	(−)	(N/m^2^ × 10^2^)	(N/m^2^ × 10^4^)	(−)	(%)	(−)	(N/m^2^ × 10^2^)	(N/m^2^ × 10^4^)	(−)
Koshihikari	–	17.8 ± 2.1	5.5 ± 0.6	79.0 ± 1.1	25.8 ± 3.4	19.3 ± 1.2	13.4 ± 1.3	19.0 ± 1.7	80.0 ± 2.1	32.4 ± 4.6	19.2 ± 3.8	21.7 ± 2.3
Takanari	–	18.2 ± 2.1	5.8 ± 0.6	**59.0 ± 1.7**	**16.3 ± 4.2**	**23.1 ± 1.7**	12.0 ± 1.2	19.6 ± 1.7	**65.0 ± 1.7**	**17.2 ± 4.7**	**22.3 ± 3.0**	20.8 ± 2.9
SL1201	Chr1	16.5 ± 1.9	5.9 ± 1.4	79.3 ± 1.0	20.0 ± 4.1	20.0 ± 1.0	11.4 ± 1.2	19.1 ± 1.5	89.0 ± 1.0	24.4 ± 5.4	20.1 ± 2.9	21.4 ± 2.8
SL1202	Chr1	15.5 ± 2.8	5.8 ± 2.1	73.7 ± 0.0	17.0 ± 3.2	17.1 ± 0.9	11.8 ± 1.3	18.6 ± 2.3	87.0 ± 0.0	20.5 ± 4.5	18.9 ± 3.2	22.4 ± 2.9
SL1203	Chr1	17.8 ± 1.9	5.9 ± 1.4	81.0 ± 1.0	22.9 ± 3.6	19.8 ± 1.0	12.3 ± 1.2	18.9 ± 1.5	83.0 ± 1.0	21.2 ± 4.8	20.2 ± 2.2	21.8 ± 2.1
SL1204	Chr1	**21.3 ± 1,7**	5.7 ± 1.4	81.7 ± 1.5	**15.6 ± 2.7**	22.0 ± 1.0	**14.9 ± 1.2**	**21.9 ± 1.5**	86.3 ± 1.5	20.9 ± 5.3	**23.3 ± 2.8**	**23.5 ± 2.5**
SL1205	Chr1	**15.6 ± 2.1**	5.6 ± 1.6	71.0 ± 1.0	17.4 ± 3.7	19.6 ± 0.8	13.9 ± 1.4	17.5 ± 1.7	82.0 ± 1.0	19.4 ± 5.7	19.3 ± 3.3	21.7 ± 2.4
SL1206	Chr2	**19.5 ± 1.7**	5.5 ± 1.4	76.3 ± 1.5	18.2 ± 4.8	19.7 ± 0.8	11.9 ± 1.1	**20.1 ± 1.5**	81.3 ± 2.5	30.0 ± 6.0	**22.0 ± 1.6**	21.8 ± 2.1
SL1207	Chr2	**15.7 ± 1.9**	5.4 ± 2.9	70.3 ± 1.7	17.0 ± 3.7	18.7 ± 1.0	11.9 ± 1.1	16.8 ± 1.5	80.0 ± 1.7	22.4 ± 5.4	18.7 ± 2.4	22.4 ± 2.4
SL1208	Chr2	16.8 ± 1.1	5.4 ± 0.8	**54.3 ± 2.6**	**9.5 ± 2.0**	18.4 ± 1.2	**10.8 ± 1.1**	17.0 ± 0.9	**68.0 ± 3.6**	**17.1 ± 2.4**	18.5 ± 1.5	–
SL1209	Chr2	16.8 ± 2.1	**4.9 ± 1.6**	**67.0 ± 1.5**	18.0 ± 3.7	18.9 ± 0.9	**10.8 ± 1.0**	16.9 ± 1.7	81.7 ± 3.5	24.7 ± 4.5	19.0 ± 1.6	20.3 ± 0.0
SL1210	Chr3	17.4 ± 2.8	5.4 ± 0.6	**69.7 ± 0.6**	**15.1 ± 3.1**	21.0 ± 1.3	13.6 ± 1.6	17.1 ± 2.3	**73.7 ± 0.6**	**18.3 ± 5.1**	19.4 ± 2.4	20.9 ± 2.0
SL1211	Chr3	17.9 ± 3.2	5.9 ± 0.9	74.0 ± 1.5	19.0 ± 3.8	20.8 ± 1.0	11.9 ± 1.3	19.1 ± 2.6	82.7 ± 1.5	20.2 ± 4.2	20.5 ± 1.9	21.0 ± 2.4
SL1212	Chr3	16.8 ± 1.1	5.4 ± 0.8	79.7 ± 0.0	17.0 ± 4.3	19.3 ± 1.3	**9.9 ± 1.1**	18.6 ± 0.9	87.0 ± 0.0	19.6 ± 4.3	19.0 ± 2.8	20.6 ± 2.2
SL1213	Chr3	16.7 ± 1.9	5.4 ± 1.4	**68.3 ± 1.4**	**14.0 ± 2.5**	19.2 ± 1.1	**14.3 ± 1.5**	18.5 ± 1.5	79.3 ± 1.5	**15.9 ± 4.7**	19.6 ± 2.2	22.5 ± 2.5
SL1214	Chr4	**16.0 ± 1.1**	5.4 ± 0.8	77.7 ± 0.6	21.6 ± 4.7	**22.2 ± 1.1**	13.1 ± 1.3	17.8 ± 0.9	85.7 ± 0.6	22.3 ± 4.2	20.0 ± 3.2	**23.6 ± 2.1**
SL1215	Chr4	17.4 ± 1.1	5.5 ± 0.8	75.7 ± 0.6	21.5 ± 4.0	**22.1 ± 1.1**	12.5 ± 1.1	19.2 ± 0.9	85.7 ± 0.6	28.2 ± 6.4	**23.6 ± 2.7**	22.5 ± 2.0
SL1216	Chr4	18.1 ± 1.9	5.5 ± 1.4	79.3 ± 1.2	24.1 ± 4.2	21.7 ± 1.1	**14.3 ± 1.3**	19.6 ± 1.5	82.3 ± 1.2	26.9 ± 5.6	21.4 ± 2.5	22.9 ± 2.6
SL1217	Chr4	18.8 ± 2.8	5.3 ± 3.6	**63.0 ± 1.5**	17.7 ± 4.8	**22.1 ± 1.5**	**14.8 ± 1.5**	19.6 ± 2.3	**73.3 ± 1.5**	30.8 ± 6.0	**23.9 ± 2.4**	**23.2 ± 2.3**
SL1218	Chr5	18.2 ± 2.1	**6.1 ± 1.6**	78.0 ± 0.6	20.1 ± 3.9	20.7 ± 0.9	12.0 ± 1.1	**20.5 ± 1.7**	84.3 ± 0.6	29.0 ± 6.2	**22.8 ± 3.0**	22.3 ± 2.1
SL1219	Chr5	**19.0 ± 3.2**	6.0 ± 0.9	73.0 ± 1.2	**16.2 ± 2.8**	21.7 ± 1.0	**14.4 ± 1.2**	**20.4 ± 2.6**	**73.3 ± 1.2**	**18.6 ± 5.6**	21.4 ± 3.5	**23.1 ± 1.9**
SL1220	Chr5	18.1 ± 3.9	5.4 ± 0.5	73.7 ± 1.2	19.1 ± 3.5	19.7 ± 1.3	13.1 ± 1.2	19.5 ± 3.1	81.3 ± 1.2	22.1 ± 3.9	**23.1 ± 2.8**	22.0 ± 2.2
SL1221	Chr6	17.7 ± 1.9	5.8 ± 0.6	73.3 ± 1.5	16.5 ± 3.1	18.3 ± 1.1	12.4 ± 1.1	18.5 ± 1.5	83.3 ± 1.5	23.5 ± 4.5	**23.1 ± 2.9**	21.5 ± 2.1
SL1222	Chr6	16.5 ± 3.9	5.5 ± 1.4	**69.0 ± 0.0**	16.5 ± 2.6	19.2 ± 1.3	**16.2 ± 1.4**	18.7 ± 3.1	80.0 ± 0.0	21.5 ± 3.5	19.1 ± 2.0	**24.2 ± 2.1**
SL1223	Chr6	**15.8 ± 3.2**	5.6 ± 0.6	76.7 ± 2.1	18.2 ± 3.9	18.7 ± 1.0	12.3 ± 1.1	18.8 ± 2.6	85.7 ± 2.1	21.0 ± 4.5	19.6 ± 2.0	22.0 ± 2.6
SL1224	Chr6	**15.8 ± 2.8**	5.5 ± 2.1	75.0 ± 1.2	23.1 ± 3.7	21.5 ± 1.1	**10.4 ± 1.5**	18.2 ± 2.3	84.7 ± 1.2	25.5 ± 4.9	21.5 ± 1.8	22.2 ± 2.1
SL1225	Chr7	18.7 ± 3.2	5.5 ± 0.9	80.0 ± 0.5	17.9 ± 3.4	21.8 ± 0.9	**9.6 ± 1.4**	**20.5 ± 2.6**	87.3 ± 1.5	19.9 ± 3.1	21.5 ± 2.3	20.7 ± 2.9
SL1226	Chr7	17.0 ± 2.1	5.4 ± 0.7	78.0 ± 1.0	18.5 ± 3.3	20.2 ± 0.8	12.3 ± 1.2	19.0 ± 1.7	88.0 ± 1.0	22.4 ± 3.8	20.3 ± 2.6	22.4 ± 2.3
SL1227	Chr7	17.5 ± 1.1	5.7 ± 0.8	76.7 ± 1.0	22.2 ± 3.3	20.0 ± 1.4	11.7 ± 1.0	18.4 ± 0.9	82.7 ± 2.5	26.2 ± 3.7	**23.0 ± 1.9**	21.5 ± 2.6
SL1228	Chr8	17.9 ± 1.9	5.6 ± 1.4	**69.3 ± 1.2**	23.5 ± 4.7	20.7 ± 1.1	13.0 ± 0.9	18.2 ± 1.5	84.7 ± 1.2	24.3 ± 3.8	20.4 ± 2.5	21.8 ± 2.0
SL1229	Chr8	16.2 ± 1.1	5.5 ± 0.8	**68.3 ± 1.0**	17.6 ± 3.5	17.6 ± 0.7	13.5 ± 1.2	17.4 ± 0.9	86.0 ± 0.0	23.4 ± 6.2	20.7 ± 2.5	22.9 ± 2.0
SL1230	Chr8	17.2 ± 2.1	**6.1 ± 1.6**	78.0 ± 2.1	18.7 ± 3.7	18.0 ± 0.8	13.1 ± 1.2	18.8 ± 1.7	87.7 ± 2.1	26.6 ± 4.9	19.9 ± 2.3	22.7 ± 2.0
SL1231	Chr8	18.2 ± 1.9	**6.0 ± 1.4**	75.0 ± 1.0	20.8 ± 3.8	18.6 ± 0.9	12.8 ± 1.0	18.9 ± 1.5	86.0 ± 1.0	23.5 ± 6.5	20.8 ± 2.3	22.5 ± 2.0
SL1232	Chr9	15.5 ± 2.8	5.5 ± 2.1	72.0 ± 1.0	18.9 ± 2.7	17.2 ± 1.1	12.2 ± 1.2	17.2 ± 2.3	81.0 ± 2.6	22.6 ± 4.5	**16.3 ± 2.1**	23.0 ± 2.2
SL1233	Chr9	**14.3 ± 3.2**	5.9 ± 0.9	74.0 ± 2.1	**14.4 ± 3.1**	17.6 ± 0.7	11.1 ± 1.3	16.6 ± 2.6	86.7 ± 1.5	22.4 ± 5.2	17.1 ± 2.5	22.4 ± 1.9
SL1234	Chr10	16.2 ± 2.8	5.5 ± 2.9	73.3 ± 1.5	19.3 ± 3.3	19.0 ± 0.9	**10.7 ± 1.2**	16.1 ± 2.3	83.3 ± 1.5	24.4 ± 6.0	17.1 ± 3.2	21.6 ± 2.1
SL1235	Chr10	**16.0 ± 2.1**	5.5 ± 1.6	**69.7 ± 1.0**	20.0 ± 3.8	18.3 ± 1.1	11.8 ± 1.3	16.2 ± 1.7	81.0 ± 1.0	19.8 ± 5.1	**16.4 ± 3.7**	22.6 ± 2.1
SL1236	Chr11	17.9 ± 3.7	5.5 ± 2.0	75.7 ± 1.2	19.7 ± 3.7	19.8 ± 1.1	12.4 ± 1.1	17.9 ± 3.0	84.7 ± 1.2	19.4 ± 4.4	18.3 ± 2.6	21.2 ± 2.8
SL1237	Chr11	**15.4 ± 2.8**	5.5 ± 1.7	**61.0 ± 1.6**	**12.6 ± 3.6**	**22.9 ± 0.9**	12.6 ± 1.5	16.6 ± 2.3	**73.0 ± 1.0**	**16.8 ± 5.8**	**23.1 ± 1.9**	**23.3 ± 2.1**
SL1238	Chr11	16.5 ± 3.2	5.4 ± 0.9	73.3 ± 1.0	16.7 ± 3.5	19.9 ± 1.2	13.3 ± 1.3	18.5 ± 2.6	87.0 ± 1.0	25.1 ± 5.2	22.0 ± 3.3	**23.0 ± 1.9**
SL1239	Chr12	**15.8 ± 2.1**	5.8 ± 1.6	**69.3 ± 0.7**	17.0 ± 3.7	20.4 ± 1.1	12.7 ± 1.2	16.7 ± 1.7	86.3 ± 1.2	23.4 ± 4.4	21.4 ± 2.8	22.8 ± 2.0
SL1240	Chr12	16.2 ± 2.1	5.7 ± 1.6	76.0 ± 1.0	17.0 ± 3.1	19.4 ± 1.1	12.0 ± 1.4	17.6 ± 1.7	86.0 ± 1.0	27.1 ± 4.8	21.3 ± 3.2	22.2 ± 2.2
SL1241	Chr12	17.6 ± 2.8	5.6 ± 2.1	**55.0 ± 1.0**	16.3 ± 2.6	17.6 ± 0.7	12.3 ± 1.1	17.7 ± 2.3	**70.0 ± 0.0**	25.1 ± 5.2	20.6 ± 2.5	–

Bold and underlined numbers indcate significant diffferences to ‘Koshihikari’ at the 5% level by the Dunnett’s multiple comparison test

**Table 2:** QTLs for eating-quality traits on CSSLs in the ‘Koshihikari’ genetic background in 2016 and 2017

Trait	Locus name	CSSLs	Position	Flanking marker interval	Year	Additive effect
Amylose content (%)	*qAC1–1*	SL1204	Chr1	RM1196-RM7594	2016	1.8
					2017	1.5
	*qAC1–2*	SL1205		RM6648-RM6321	2016	−1.1
	*qAC2–1*	SL1206, SL1207	Chr2	RM5897-RM1234	2016	0.9
					2017	0.6
	*qAC4*	SL1214	Chr4	RM16260-RM1305	2016	−0.9
	*qAC5*	SL1219	Chr5	RM6034-RM1386	2016	0.6
					2017	0.8
	*qAC6*	SL1223, SL1224	Chr6	RM1340-RM1370	2016	−1.0
	*qAC7*	SL1225	Chr7	RM4584-RM5481	2017	0.8
	*qAC9*	SL1233	Chr9	RM6235-RM6797	2016	−1.8
	*qAC10*	SL1235	Chr10	RM4455-RM6673	2016	−0.9
	*qAC11*	SL1237	Chr11	RM3701-RM1341	2016	−1.2
	*qAC12*	SL1239	Chr12	Bb77A02-RM2935	2016	−1.0
Protein content (%)	*qPC2*	SL1209	Chr2	RM6933-RM3850	2016	−0.3
	*qPC5*	SL1218	Chr5	RM6034-RM1386	2016	0.3
	*qPC8*	SL1230, SL1231	Chr8	RM3634-RM4997	2016	0.3
Eating quality score (−)	*qEQ2*	SL1208, SL1209	Chr2	RM3515-RM3850	2016	−12.4
					2017	−6.0
	*qEQ3–1*	SL1210	Chr3	RM7332-RM5748	2016	−4.7
					2017	−3.2
	*qEQ3–2*	SL1213		RM2334-RM7389	2016	−5.4
	*qEQ4*	SL1217	Chr4	RM3839-RM5608	2016	−8.0
					2017	−3.4
	*qEQ5*	SL1219	Chr5	RM6034-RM1386	2017	−3.4
	*qEQ6*	SL1222	Chr6	RM5855-RM7193	2016	−5.0
	*qEQ8*	SL1228, SL1229	Chr8	RM6369-RM22709	2016	−5.4
	*qEQ10*	SL1235	Chr10	RM4455-RM6673	2016	−4.7
	*qEQ11*	SL1237	Chr11	RM3701-RM1341	2016	−9.0
					2017	−3.5
	*qEQ12–1*	SL1239	Chr12	Bb77A02-RM2935	2016	−4.9
	*qEQ12–2*	SL1241		RM3326-RM1226	2016	−12.0
					2017	−5.0
Stickiness S1 (N/m^2^ × 10^2^)	*qST1*	SL1204	Chr1	RM1196-RM7594	2016	−5.1
	*qST2*	SL1208	Chr2	RM1211-RM3316	2016	−8.2
					2017	−7.7
	*qST3–1*	SL1210	Chr3	RM7332-RM5748	2016	−5.4
					2017	−7.1
	*qST3–2*	SL1213		RM2334-RM7389	2016	−5.9
					2017	−8.3
	*qST5*	SL1219	Chr5	RM6034-RM1386	2016	−4.8
					2017	−6.9
	*qST9*	SL1233	Chr9	RM6235-RM6797	2016	−5.7
	*qST11*	SL1237	Chr11	RM3701-RM1341	2016	−6.6
					2017	−7.8
Hardness H2 (N/m^2^ × 10^4^)	*qHA1*	SL1204	Chr1	RM1196-RM7594	2017	2.1
	*qHA2*	SL1206	Chr2	RM6842-RM5699	2017	1.4
	*qHA4–1*	SL1214, SL1215	Chr4	RM5414-RM5633	2016	1.5
					2017	2.2
	*qHA4–2*	SL1217		RM3839-RM5608	2016	1.4
					2017	2.4
	*qHA5–1*	SL1218	Chr5	RM1248-RM3838	2017	1.8
	*qHA5–2*	SL1220		RM1386-RM3286	2017	2.0
	*qHA6–1*	SL1221	Chr6	RM6467-RM5855	2017	2.0
	*qHA7*	SL1227	Chr7	RM6394-RM7601	2017	1.9
	*qHA9*	SL1232	Chr9	RM23654-RM6235	2017	−1.5
	*qHA10*	SL1235	Chr10	RM4455-RM6673	2017	−1.4
	*qHA11*	SL1237	Chr11	RM3701-RM1341	2016	1.8
					2017	2.0
Whiteness (−)	*qWH1*	SL1204	Chr1	RM1196-RM7594	2016	0.8
					2017	0.9
	*qWH2–1*	SL1208, SL1209	Chr2	RM3515-RM3850	2016	−1.3
	*qWH3*	SL1212, SL1213	Chr3	RM3513-RM6970	2016	0.5
	*qWH4–1*	SL1214	Chr4	RM16260-RM1305	2017	1.0
	*qWH4–2*	SL1216, SL1217		RM1359-RM3916	2016	0.7
					2017	0.8
	*qWH5*	SL1219	Chr5	RM6034-RM1386	2016	0.5
					2017	0.7
	*qWH6–1*	SL1222	Chr6	RM5855-RM7193	2016	1.4
					2017	1.3
	*qWH6–2*	SL1224		RM5957-RM5463	2016	−1.5
	*qWH7*	SL1225	Chr7	RM4584-RM5481	2016	−1.9
	*qWH10*	SL1234	Chr10	RM7492-RM1859	2016	−1.4
	*qWH11*	SL1237, SL1238	Chr11	RM5824-RM6623	2017	0.8

Positive additive effect means ‘Takanari’ allele increasing the trait values

**Fig. 1:**
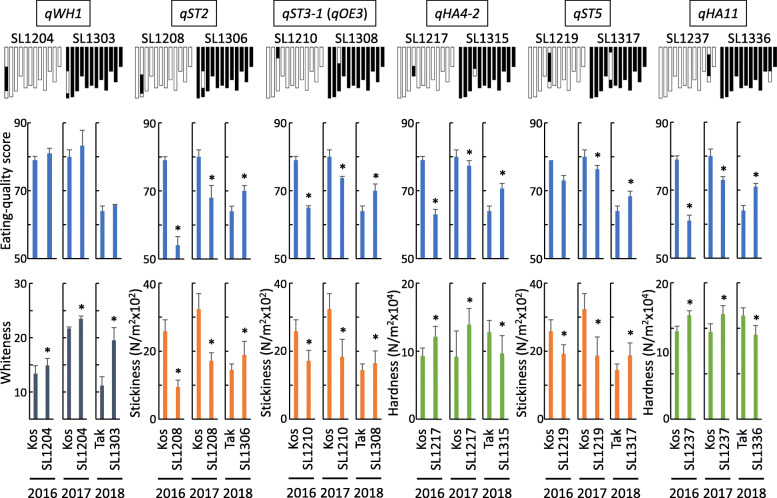
Eating quality score, stickiness of the surface of cooked rice grains, hardness of whole cooked rice grains and whiteness of rice grains in 12 chromosome segment substitution lines carrying single introduced segments on chromosomes 1–5 or 11 in the ‘Koshihikari’ (Kos) genetic background in 2016 and 2017, and in the ‘Takanari’ (Tak) genetic background in 2018. White bars, homozygous for the ‘Koshihikari’ allele; black bars, homozygous for the ‘Takanari’ allele. Data for eating quality traits are presented as means ± SD (*n* = 6). Asterisks indicate significant differences in each eating quality traits between CSSLs and the recurrent cultivars ‘Koshihikari’ or ‘Takanari’ at *P* < 0.05 by the Dunnet’s test

We focused on six of the detected QTLs (Fig. 1): *qWH1* for grain whiteness on the long arm of chromosome 1, *qST2* for stickiness of cooked grains on the long arm of chromosome 2, *qST3–1* for stickiness of cooked grains on the short arm of chromosome 3, *qHA4–2* for hardness of cooked grains on the long arm of chromosome 4, *qST5* for stickiness of cooked grains on the long arm of chromosome 5 and *qHA11* for hardness of cooked grains on the long arm of chromosome 11. We selected these six QTLs because of their large genetic effects and detection in both years.

In comparison with ‘Koshihikari’, SL1204, carrying *qWH1*, had significantly higher whiteness, amylose content and hardness of cooked grains, but lower stickiness of cooked grains in 2016 and 2017. SL1208, carrying *qST2*, had significantly lower eating quality score, stickiness of cooked grains and grain whiteness. SL1210, carrying *qST3–1*, had significantly lower eating quality score and stickiness of cooked grains. SL1217, carrying *qHA4–2*, had significantly lower eating quality score and higher hardness of cooked grains and grain whiteness. SL1219, carrying *qST5*, had significantly lower eating quality score and stickiness of cooked grains but higher amylose content and grain whiteness. SL1237, carrying *qHA11*, had significantly lower eating quality score, stickiness of cooked grains and amylose content, hardness of cooked grains and grain whiteness. The ‘Takanari’ allele of *qWH1* increased grain whiteness. The ‘Koshihikari’ alleles of the remaining five QTLs resulted in high eating quality score and in strong stickiness and softness of cooked grains. *qST2*, *qST3–1* and *qHA4–2* were not associated with differences in amylose or protein contents in both years.

Eating quality traits are easily affected by many agronomic traits including heading date, grain size and weight, grain number per panicle and spikelet fertility (Juliano et al. 1993; Iijima et al. 2019). We evaluated agronomic traits of the CSSLs, in particular those of the six CSSLs each carrying a QTL on chromosomes 1–5 and 11. In comparison with ‘Koshihikari’, five lines showed no significant differences in days to heading (flowering time), culm length, panicle length, number of panicles, unhulled grain weight or spikelet fertility (Supplementary Tables S2 and S3). However, one line, SL1208, showed weak vigor, including few panicles and low unhulled grain weight and spikelet fertility.

### QTLs for Eating Quality Traits in CSSLs of the ‘Takanari’ Genetic Background

Among the CSSLs of the ‘Takanari’ genetic background, there was also a wide range of phenotypic differences in eating quality traits (Table 3, Supplementary Table S4, Supplementary Figure 1). In comparison with ‘Takanari’, 36 QTLs in 2018 showed significant differences in the six eating quality traits (Table 4). The six QTLs detected in the ‘Koshihikari’-background CSSLs on chromosomes 1–5 and 11 coincided well with those detected in the ‘Takanari’ CSSLs (Fig. 1).

**Table 3:** Eating-quality traits on CSSLs in the ‘Takanari’ genetic background in 2018

		2018					
				Cooked Rice Taste Analyzer	Tensipresser		Rice Grain Image Analyzer
		Amylose content	Protein content	Eating quality score	Stickiness S1	Hardness H2	Whiteness
Line No.	Donor segment	(%)	(%)	(−)	(N/m^2^ × 10^2^)	(N/m^2^ × 10^4^)	(−)
Takanari	–	15.8 ± 2.3	5.7 ± 1.1	64.0 ± 1.5	14.4 ± 4.0	22.8 ± 1.7	11.2 ± 1.6
Koshihikari	–	15.0 ± 2.3	5.1 ± 1.1	**84.3 ± 1.0**	**20.5 ± 3.1**	19.4 ± 1.2	14.7 ± 2.6
SL1301	Chr1	17.0 ± 2.0	5.9 ± 1.0	67.3 ± 1.2	13.5 ± 2.6	20.9 ± 1.6	14.3 ± 2.1
SL1302	Chr1	16.8 ± 3.0	5.5 ± 1.5	**74.0 ± 1.6**	14.7 ± 3.3	23.0 ± 1.3	15.7 ± 2.2
SL1303	Chr1	13.7 ± 2.0	5.9 ± 1.0	66.0 ± 1.3	12.8 ± 2.7	22.0 ± 1.0	**19.5 ± 2.3**
SL1304	Chr1	**18.0 ± 2.0**	5.5 ± 1.0	69.7 ± 2.1	14.6 ± 1.2	21.8 ± 1.7	14.5 ± 2.5
SL1305	Chr2	14.5 ± 2.3	5.5 ± 1.1	67.0 ± 1.7	14.3 ± 2.5	20.2 ± 1.1	14.3 ± 2.7
SL1306	Chr2	16.5 ± 2.0	5.5 ± 1.0	**70.0 ± 1.6**	**16.8 ± 2.4**	19.0 ± 1.2	**16.3 ± 2.1**
SL1307	Chr2	16.7 ± 2.0	5.4 ± 1.0	69.0 ± 1.7	12.0 ± 3.4	21.7 ± 1.4	13.6 ± 2.6
SL1308	Chr3	16.8 ± 1.2	5.5 ± 0.6	**70.1 ± 2.0**	**16.6 ± 3.6**	20.2 ± 1.6	15.0 ± 2.4
SL1309	Chr3	16.4 ± 2.3	5.2 ± 1.1	66.3 ± 1.5	15.5 ± 3.8	22.6 ± 1.5	15.5 ± 2.3
SL1310	Chr3	14.3 ± 2.0	5.4 ± 1.5	67.7 ± 1.5	16.5 ± 3.3	20.5 ± 1.4	13.5 ± 2.9
SL1311	Chr3	**19.2 ± 2.5**	5.9 ± 1.7	68.7 ± 1.2	16.5 ± 4.6	23.3 ± 1.6	**16.8 ± 2.7**
SL1312	Chr4	16.3 ± 1.2	5.4 ± 0.6	65.7 ± 2.1	12.6 ± 2.6	**19.0 ± 1.4**	**16.2 ± 2.2**
SL1313	Chr4	15.4 ± 2.0	5.4 ± 1.0	64.7 ± 1.5	14.7 ± 3.2	22.0 ± 1.7	15.3 ± 1.9
SL1314	Chr4	16.3 ± 1.2	5.4 ± 0.6	69.3 ± 1.2	16.0 ± 3.7	22.4 ± 1.4	14.6 ± 2.6
SL1315	Chr4	15.6 ± 1.2	5.5 ± 0.6	**70.7 ± 1.5**	13.3 ± 0.6	**18.7 ± 2.6**	13.2 ± 2.5
SL1316	Chr5	17.6 ± 2.0	5.5 ± 1.0	64.7 ± 2.3	13.3 ± 3.7	**25.1 ± 1.5**	15.2 ± 1.9
SL1317	Chr5	14.5 ± 3.0	**6.3 ± 1.0**	**71.3 ± 1.5**	**18.7 ± 3.7**	**17.4 ± 1.1**	12.3 ± 2.2
SL1318	Chr5	14.7 ± 2.3	6.0 ± 1.1	68.3 ± 1.5	13.4 ± 3.4	22.1 ± 1.0	12.1 ± 2.0
SL1319	Chr6	17.2 ± 3.5	6.0 ± 1.7	68.3 ± 2.1	14.0 ± 2.1	21.8 ± 3.0	15.1 ± 2.2
SL1320	Chr6	–	–	–	–	–	–
SL1321	Chr6	17.3 ± 2.0	5.8 ± 1.0	69.7 ± 3.1	12.9 ± 3.2	20.1 ± 1.3	12.8 ± 2.4
SL1322	Chr6	16.6 ± 4.1	5.6 ± 2.0	65.7 ± 1.5	14.5 ± 2.6	**18.7 ± 1.4**	**9.4 ± 2.2**
SL1323	Chr7	15.5 ± 3.5	5.5 ± 1.7	**70.7 ± 1.5**	12.6 ± 2.8	19.9 ± 2.2	**16.2 ± 3.2**
SL1324	Chr7	17.6 ± 3.0	5.4 ± 1.5	–	13.4 ± 3.0	22.0 ± 1.6	**18.2 ± 3.4**
SL1325	Chr7	13.5 ± 3.5	5.2 ± 1.7	**76.0 ± 0.6**	15.9 ± 2.8	19.0 ± 1.2	13.9 ± 2.7
SL1326	Chr8	16.1 ± 2.3	5.4 ± 1.1	68.3 ± 0.6	15.9 ± 1.8	20.7 ± 1.8	15.9 ± 2.1
SL1327	Chr8	15.5 ± 1.2	5.8 ± 0.6	–	14.1 ± 2.6	21.0 ± 1.4	14.0 ± 2.5
SL1328	Chr8	15.0 ± 2.0	5.6 ± 1.0	62.0 ± 1.6	13.7 ± 2.7	**17.0 ± 1.7**	12.8 ± 3.1
SL1329	Chr9	14.8 ± 1.2	5.5 ± 0.6	57.3 ± 1.2	14.9 ± 2.8	**25.5 ± 1.4**	13.3 ± 1.9
SL1330	Chr9	14.6 ± 2.3	**6.1 ± 1.1**	63.3 ± 2.3	12.1 ± 2.8	22.3 ± 1.5	14.9 ± 2.3
SL1331	Chr9	**18.4 ± 2.0**	6.0 ± 1.0	58.7 ± 2.0	**9.8 ± 1.7**	22.9 ± 1.5	12.0 ± 2.0
SL1332	Chr10	16.4 ± 3.0	5.5 ± 1.5	68.0 ± 2.0	11.5 ± 2.3	23.3 ± 1.9	15.3 ± 2.1
SL1333	Chr10	15.0 ± 3.5	5.9 ± 1.7	**71.0 ± 1.6**	13.3 ± 2.2	21.0 ± 1.3	14.5 ± 2.8
SL1334	Chr10	**18.2 ± 3.0**	5.5 ± 1.5	**71.0 ± 2.6**	13.7 ± 3.3	23.9 ± 1.7	14.3 ± 2.8
SL1335	Chr11	14.8 ± 2.3	5.5 ± 1.1	60.0 ± 1.5	**17.8 ± 2.0**	22.3 ± 1.9	15.3 ± 3.2
SL1336	Chr11	**18.3 ± 2.0**	**6.1 ± 1.0**	**74.0 ± 1.6**	**17.4 ± 1.8**	**18.6 ± 2.0**	13.9 ± 2.0
SL1337	Chr12	**18.6 ± 2.0**	5.5 ± 1.5	66.3 ± 2.3	14.3 ± 2.5	21.5 ± 1.7	12.4 ± 1.8
SL1338	Chr12	14.6 ± 3.5	5.4 ± 1.7	65.3 ± 0.6	11.9 ± 2.3	21.1 ± 0.9	13.4 ± 2.2
SL1339	Chr12	16.5 ± 2.3	5.8 ± 1.1	64.7 ± 0.6	13.6 ± 2.7	22.5 ± 1.8	14.5 ± 2.9

Bold and underlined numbers indcate significant diffferences to ‘Koshihikari’ at the 5% level by the Dunnett’s multiple comparison tes

**Table 4:** QTLs for eating-quality traits on CSSLs in the ‘Takanari’ genetic background in 2018

Trait	Locus name	CSSLs	Position	Flanking marker interval	Additive effect
Amylose content (%)	*qAC1–2*	SL1304	Chr1	*sd1*-RM6321	1.1
	*qAC3*	SL1311	Chr3	RM2334-RM7389	1.7
	*qAC9*	SL1331	Chr9	RM5657-RM6797	1.3
	*qAC10*	SL1334	Chr10	RM4455-RM6673	1.2
	*qAC11*	SL1336	Chr11	RM1355-RM7443	1.3
	*qAC12*	SL1337	Chr12	Bb77A02-RM7102	1.4
Protein content (%)	*qPC5*	SL1317	Chr5	RM6034-RM3476	0.3
	*qPC9*	SL1330	Chr9	RM3907-RM6235	0.2
	*qPC11*	SL1336	Chr11	RM1355-RM7443	0.2
Eating quality score (−)	*qEQ1*	SL1302	Chr1	RM1287-RM1297	−5.0
	*qEQ2*	SL1306	Chr2	RM5699-RM1379	−3.0
	*qEQ3–1*	SL1308	Chr3	RM7332-RM5748	−3.1
	*qEQ4*	SL1315	Chr4	RM3839-RM5608	−3.4
	*qEQ5*	SL1317	Chr5	RM6034-RM3476	−3.7
	*qEQ7–1*	SL1323	Chr7	RM4584-RM6728	−3.4
	*qEQ7–2*	SL1325		RM6394-RM7601	−6.0
	*qEQ10*	SL1333, SL1334	Chr10	RM5348-RM5620	−3.5
	*qEQ11*	SL1336	Chr11	RM1355-RM7443	−5.0
Stickiness S1 (N/m^2^ × 10^2^)	*qST2*	SL1306	Chr2	RM5699-RM1379	−1.2
	*qST3–1*	SL1308	Chr3	RM7332-RM5748	−1.1
	*qST5*	SL1317	Chr5	RM6034-RM3476	−2.2
	*qST9*	SL1331	Chr9	RM5657-RM6797	2.3
	*qST11*	SL1335, SL1336	Chr11	RM5824-RM6623	−1.7
Hardness H2 (N/m^2^ × 10^4^)	*qHA4–1*	SL1312	Chr4	RM16260-RM5633	1.9
	*qHA4–2*	SL1315		RM3839-RM5608	2.1
	*qHA5–1*	SL1316, SL1317	Chr5	RM17836-RM18222	2.7
	*qHA6–2*	SL1322	Chr6	RM5957-RM5463	2.1
	*qHA8*	SL1328	Chr8	RM5767-RM4997	2.9
	*qHA9*	SL1329	Chr9	RM23654-RM3907	−1.4
	*qHA11*	SL1336	Chr11	RM1355-RM7443	2.1
Whiteness (−)	*qWH1*	SL1303	Chr1	RM7124-*sd1*	4.2
	*qWH2–2*	SL1306	Chr2	RM5699-RM1379	2.6
	*qWH3*	SL1311	Chr3	RM2334-RM7389	2.8
	*qWH4–1*	SL1312	Chr4	RM16260-RM5633	2.5
	*qWH6–2*	SL1322	Chr6	RM5957-RM5463	−0.9
	*qWH7*	SL1324, SL1325	Chr7	RM5481-RM3826	3.5

Positive additive effect means ‘Takanari’ allele increasing the trait values

In comparison with ‘Takanari’, SL1303, carrying *qWH1*, had significantly higher grain whiteness. Thus, this chromosome region increased whiteness both in the ‘Koshihikari’ and ‘Takanari’ genetic backgrounds. SL1306, carrying *qST2*, had significantly higher eating quality score, stickiness of cooked grains and grain whiteness. SL1308, carrying *qST3–1*, had significantly higher eating quality score and grain stickiness. SL1315, carrying *qHA4–2*, had significantly higher eating quality score and lower hardness of cooked grains. SL1317, carrying *qST5*, had significantly higher eating quality score, stickiness of cooked grains and protein content, and lower hardness of cooked grains. SL1336, carrying *qHA11*, had significantly higher eating quality score, amylose content, protein content and grain stickiness, and lower hardness of cooked grains. The ‘Koshihikari’ alleles of all of the six QTLs resulted in high eating quality score, strong stickiness and softness of cooked grains, and high grain whiteness in the ‘Takanari’ genetic background. *qST2*, *qST3–1* and *qHA4–2* were not associated with significant differences in amylose or protein contents.

We also evaluated agronomic traits in the CSSLs of the ‘Takanari’ genetic background. In comparison with ‘Takanari’, SL1303, SL1306, SL1308, SL1315 and SL1317 showed no significant differences in days to heading, culm length, panicle length, number of panicles or unhulled grain weight (Supplementary Table S4). However, SL1336 showed weak vigor including late flowering, short culms and panicles, few panicles and low unhulled grain weight.

### Confirmation of the Effects of QTLs in CSSLs of Other *indica* Cultivars

**Fig. 2:**
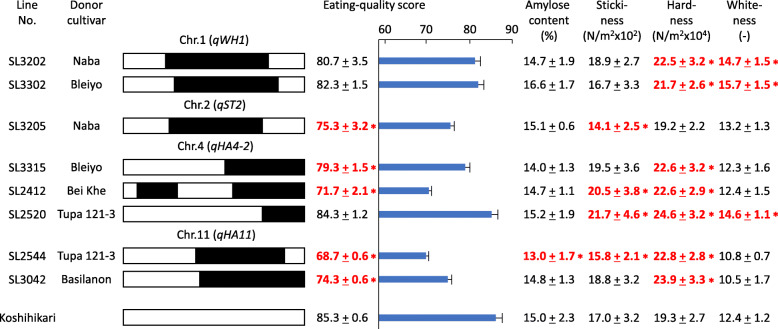
Eating quality traits and genotypes of eight CSSLs derived from crosses between the recurrent parent ‘Koshihikari’ and donor *indica* rice cultivars. The short arm is on the left and the long arm on the right. White bars, homozygous for the ‘Koshihikari’ allele; black bars, homozygous for the donor cultivar allele. Data for eating quality traits are presented as means ± SD (n = 6). Red numbers and asterisks indicate significant differences in eating quality traits between CSSLs and ‘Koshihikari’ at *P* < 0.05 by the Dunnet’s test

To investigate whether the six detected QTLs would be commonly detected in segregating populations derived from crosses between *japonica* and other *indica* rice cultivars, we developed additional CSSLs carrying chromosome segments introduced from *indica* cultivars ‘Naba’, ‘Bleiyo’, ‘Bei Khe’, ‘Tupa 121–3’ and ‘Basilanon’ in the ‘Koshihikari’ genetic background. Many of the eating quality traits of these CSSLs were significantly different from those of ‘Koshihikari’ (Fig. 2, Supplementary Table S5).

SL3202 and SL3302 each had a single segment on the long arm of chromosome 1 derived from ‘Naba’ and ‘Bleiyo’, respectively. In both lines, grain whiteness was significantly higher than in ‘Koshihikari’. High whiteness in SL3202 and SL3302 was the same phenotype as that of SL1204 carrying *qWH1* from ‘Takanari’ (Fig. 1). Eating quality scores of SL3202 and SL3302 were not significantly different from that of ‘Koshihikari’. In comparison with ‘Koshihikari’, hardness of cooked grains was higher in SL3202 and SL3302, as it was in SL1204 in 2017. SL3205 had a single segment on the long arm of chromosome 2 derived from ‘Naba’. The eating quality score and stickiness of cooked grains were significantly lower in SL3205 than in ‘Koshihikari’. These phenotypes were the same as that of SL1208 carrying *qST2* from ‘Takanari’. SL3315, SL2412 and SL2520 each had chromosome segments on the long arm of chromosome 4 derived from ‘Bleiyo’, ‘Bei Khe’ and ‘Tupa 121–3’, respectively. SL3315 showed significantly lower eating quality score and higher hardness of cooked grains than those of ‘Koshihikari’. SL2412 had a significantly lower eating quality score, but higher stickiness and hardness of cooked rice grains. SL2520 showed significantly higher grain whiteness and stickiness and hardness of cooked grains, but its eating quality score was not significantly different from that of ‘Koshihikari’. The phenotypes of SL3315, SL2412 and SL2520 (low eating quality score and stickiness, and high hardness of cooked grains) were similar to those of SL1217, which had *qHA4–2* from ‘Takanari’. SL2544 and SL3042 each had a single segment on the long arm of chromosome 11 derived from ‘Tupa 121–3’ and ‘Basilanon’, respectively. SL2544 had lower eating quality score, amylose content and stickiness of cooked grains, and higher hardness of cooked grains. SL3042 had lower eating quality score and higher hardness of cooked grains. These phenotypes were similar to those of SL1237, which had *qHA11* from ‘Takanari’.

The QTLs in the CSSLs derived from crosses with other *indica* rice cultivars were detected in the same chromosome regions as *qWH1*, *qST2*, *qHA4–2* and *qHA11*, confirming the genetic effects of these QTLs not only in ‘Takanari’ but also in several other *indica* cultivars. All of the CSSLs carrying *qWH1*, *qST2*, *qHA4–2* and *qHA11* except SL2544 showed no significant differences from ‘Koshihikari’ in amylose contents.

## Discussion

### QTLs for Improving Eating Quality in *indica* Rice Cultivars

There is a wide range of phenotypic variations in eating quality traits among rice cultivars, and consumer preferences differ considerably worldwide (Juliano et al. 1993; Calingacion et al. 2014). Eating quality traits are very different even within *indica* and *japonica* rice (Calingacion et al. 2014; Hori et al. 2016; Iijima et al. 2019). Generally, cooked grains of *japonica* cultivars are more sticky and softer than those of *indica* cultivars (Hori and Yano 2013). In the cultivation areas of *japonica* cultivars such as China, Korea and Japan, *indica* cultivars are often evaluated to have lower eating quality than typical *japonica* cultivars. In this study, we detected six QTLs for eating quality traits involved in differences between *indica* and *japonica* rice cultivars using a reciprocal set of CSSLs derived from a cross between a *japonica* cultivar ‘Koshihikari’ and an *indica* cultivar ‘Takanari’.

One eating quality QTL, *qOE3*, has been commonly detected on the short arm of chromosome 3 by using mapping populations derived from crosses between Japanese *japonica* cultivars (Kobayashi and Tomita 2008; Takeuchi et al. 2008; Wada et al. 2008; Hori and Yano 2013). In these studies, the *qOE3* showed the largest genetic effect among the detected QTLs for eating quality and stickiness of cooked rice grains in the previous studies and the ‘Koshihikari’ allele of *qOE3* was associated with good eating quality and strong stickiness of cooked grains. Here, we detected one eating quality QTL, *qST3–1*, also on the short arm of chromosome 3, and the ‘Koshihikari’ allele of this QTL was also associated with high eating quality score and strong stickiness of cooked grains. The ‘Takanari’ allele of *qOE3* would be the same as the ‘Nipponbare’ allele because of consistent haplotypes between these cultivars according to the RAP-DB and TASUKE databases (Sakai et al. 2013; Kawahara et al. 2013; Kumagai et al. 2019). Therefore, *qST3–1* is likely identical to *qOE3*.

**Fig. 3:**
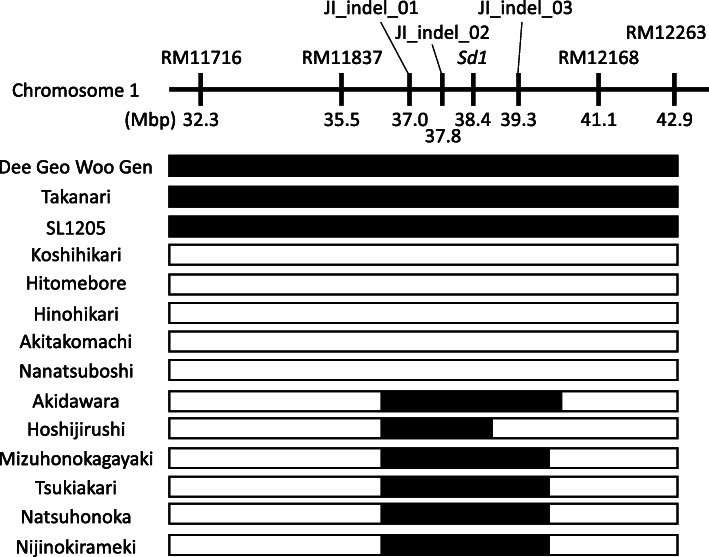
Genotypes near the *sd1* gene on the long arm of chromosome 1 in ‘Dee-Geo-Woo-Gen’, ‘Takanari’, SL1205, five Japanese leading cultivars and six high-yielding cultivars released since 2011. The centromere side is on the left, and the distal end of the long arm is on the right. White bars, homozygous for the ‘Koshihikari’-type allele; black bars, homozygous for the ‘Takanari’-type allele

We detected five other QTLs with large genetic effects on chromosomes 1, 2, 4, 5 and 11. Both ‘Takanari’ and ‘Koshihikari’ segments of the long arm of chromosome 1 containing *qWH1* resulted in high whiteness and high eating quality score in the ‘Koshihikari’ and ‘Takanari’ genetic backgrounds, respectively. We confirmed the genetic effects of *qWH1* in two additional CSSLs carrying chromosome segments derived from *indica* cultivars ‘Naba’ and ‘Bleiyo’. These data suggest the presence of at least two distinct QTLs for increasing grain whiteness on the long arm of chromosome 1 in *japonica* and *indica* rice cultivars. To investigate importance of *qWH1* in Japanese rice breeding programs, we investigated genotypes of the *qWH1* region in Japanese leading rice cultivars and recently developed rice cultivars (Fig. 3). In the six recently released cultivars, a genome sequence between 37.0 and 39.3 Mbp of the *qWH1* region containing the *sd1* gene is replaced with *indica*-type chromosome segments, while the same region in the five leading Japanese cultivars is of *japonica* type. This difference may be caused not only by selection of semi-dwarf phenotypes caused by the *sd1* gene during breeding, but also by selection of grain whiteness caused by *qWH1*. We cannot be certain whether the QTLs detected in multiple *indica* cultivars are the same or correspond to different genes. Further genetic analysis, including fine mapping of *qWH1*, is needed.

The ‘Koshihikari’ alleles of *qST2*, *qHA4–2*, *qST5* and *qHA11* on the long arms of chromosomes 2, 4, 5 and 11, respectively, improved eating quality traits by increasing stickiness and softening cooked rice grains. There were several reports of QTL detection in mapping populations with the same *Wx*^*b*^ allele derived from crosses between *japonica* cultivars. Kwon et al. (2011) found one QTL for eating quality and glossiness of cooked grains on the long arm of chromosome 4. Park et al. (2019) also detected one QTL for eating quality and glossiness of cooked grains on the long arm of chromosome 4, and they fine-mapped another eating quality QTL on the long arm of chromosome 9. Hsu et al. (2014) and Xu et al. (2016) reported QTLs for palatability of cooked grains detected by QTL analysis and genome-wide association study, respectively, in *japonica* rice cultivars. They reported coexistence of several QTLs and starch biosynthesis genes in the same chromosome regions. Kinoshita et al. (2017) detected three amylose content QTLs and eight protein content QTLs on chromosomes 1–4, 6, 8, 9 and 12. Takemoto-Kuno et al. (2015) found one amylose content QTL near the centromeric region on the long arm of chromosome 2. These QTLs might not be the same as those detected in our present study, because of difference in chromosome locations.

The QTLs *qST2*, *qST3–1* and *qHA4–2* on chromosomes 2, 3 and 4 were not associated with differences in amylose or protein contents. Eating quality traits are quantitative and complex, and are associated with various factors including grain composition and stickiness, hardness and whiteness of cooked grains. In this study, we found QTLs responsible for each of these factors. And, these QTLs might be eating quality genes with different molecular functions from those of previously isolated genes such as *Wx*, *Alk* and other starch biosynthesis genes, or storage protein genes. Generally, differences in eating quality between *japonica* and *indica* rice cultivars seem to be primarily due to different amylose contents caused by allelic differences of the *Wx* and *Alk* genes. Many studies have reported QTLs for eating quality and starch characteristics corresponding to the *Wx* and *Alk* genes in mapping populations derived from crosses between *indica* and *japonica* rice cultivars (Tan et al. 1999; Wan et al. 2004; Tian et al. 2005; Takeuchi et al. 2007; Su et al. 2011; Yang et al. 2018; Yang et al. 2020). The ‘Takanari’ allele of the *Wx* gene is identical with the *japonica*-type *Wx*^*b*^ allele, and the amylose content is not much different from that of ‘Koshihikari’ (Aoki et al. 2015; Hori et al. 2016; Iijima et al. 2019). In this study, we did not also detect any QTLs in the chromosome region of the *Alk* gene, which also greatly affects starch properties. ‘Takanari’ has A-type in the G / A polymorphism and TT-type in the GC / TT polymorphism of the *Alk* gene based on genotyping by the DNA marker of Bao et al. (2006) and Hiratsuka et al. (2010). It was different with other weak functional alleles of the *japonica*-type *alk* (*Alk*^*a*^) in ‘Koshihikari’, *Alk* (*Alk*^*c*^) in typical *indica* type and *Alk*^*b*^ in the previous study (Chen et al. 2020), but the same weak functional allele as other *japonica* rice cultivars such as ‘Asahi’ and ‘Akebono’ according to the RAP-DB and Rice-TASUKE database (https://ricegenomes.dna.affrc.go.jp/, Sakai et al. 2013; Kawahara et al. 2013; Kumagai et al. 2019). Therefore, ‘Koshihikari’ and ‘Takanari’ have the two weak functional alleles of both the *Wx* and *Alk* genes. This study found novel eating quality QTLs other than the *Wx* and *Alk* genes. However, we also consider other reasons for no detecting QTLs near the *Wx* and *Alk* genes. For an example, small genetic effect QTLs in this chromosome region might be concealed by other large genetic effect QTLs. Further studies are required to assess which genes or QTLs are responsible for varietal differences in eating-quality traits.

### Common Location of Detected QTLs in *indica* Rice Cultivars

The ‘Koshihikari’ alleles of all QTLs except *qWH1* improved eating quality traits. Therefore, the QTLs detected in this study could be used to improve eating quality of many *indica* cultivars. Because the effect of each single QTL did not improve eating quality to the level of typical *japonica* cultivars, it would be necessary to accumulate multiple QTL alleles to develop novel *indica* cultivars with both good eating quality and high grain yield.

Confirmation of *qST2*, *qHA4–2* and *qHA11* in CSSLs carrying chromosome segments from various *indica* cultivars suggests common allelic differences in these QTLs between *indica* and *japonica* subspecies. The difference in eating quality between *japonica* and *indica* cultivars might be due mainly to accumulation of genetic effects of QTLs detected in this study and *Wx* and *Alk* genes.

### Perspectives for Application to Future Rice Breeding

Although *indica* cultivars are considered by consumers in Northeast Asian countries such as China, Korea and Japan to have lower eating quality than typical *japonica* cultivars, many *indica* cultivars and hybrid rice cultivars have high grain yield (Cheng et al. 2007; Mackill and Khush 2018) and therefore must have genes that increase grain yield. To develop novel rice cultivars that would combine high grain yield and good (*japonica*-like) eating quality, it is necessary to combine genetic loci that improve eating quality in the background of *indica* cultivars. Global warming is expected to increase temperatures by 2 °C by the end of this century (IPCC (Intergovernmental Panel on Climate Change), 2018). Rice cultivars with good eating quality in the *indica* genetic background would be a good solution to mitigate the effects of climate warming on rice production while preserving the eating quality preferred in Northeast Asia.

However, it is often difficult to select progenies of crosses between *indica* and *japonica* rice cultivars. In modern breeding programs, the standard method includes crossing cultivars, fixing genotypes by self-pollination and selecting appropriate lines based on their phenotypes. However, many progenies derived from *indica* and *japonica* cultivars have hybrid sterility or hybrid breakdown because of differentiation between the genomes of the two subspecies and incompatibilities in many gene alleles (Matsubara et al. 2007; Yamamoto et al. 2007). If the precise chromosome positions and genetic effects of individual QTLs and genes were revealed, DNA marker technologies could allow us to improve selection efficiency in breeding when using populations derived from crosses between the subspecies. Fine-mapping of individual QTLs will be indispensable in the future. It may also be possible to reproduce favorable alleles by genome editing technology after the responsible genes are identified. Recently, we developed high-speed advanced generation technologies that use a biotron breeding system (Tanaka et al. 2016). These methods would facilitate accumulation of agronomically important genes, such as those for eating quality, grain yield, disease resistance and stress tolerance, across hybridization barriers between subspecies.

## Conclusion

We detected QTLs involved in the control of eating quality traits in CSSLs derived from a cross between a *japonica* rice cultivar ‘Koshihikari’ and an *indica* rice cultivar ‘Takanari’. Four of these QTLs, on chromosomes 1, 2, 4 and 11, were common in CSSLs derived from several other *indica* cultivars. These QTLs could be useful for improving eating quality of high-yielding *indica* cultivars to the level of typical *japonica* cultivars.

## Supplementary Information

**Additional file 1: Figure S1.** Eating quality score, amylose content, protein content, stickiness of the surface of cooked rice grains, hardness of whole cooked rice grains and whiteness of rice grains in all chromosome segment substitution lines in the ‘Koshihikari’ genetic background in 2016 (upper) and 2017 (middle), and in the ‘Takanari’ genetic background in 2018 (lower). Data for eating quality traits are presented as means ± SD (*n* = 6).

**Additional file 2: Table S1.** Graphical genotypes of eight CSSLs in the ‘Koshihikari’ genetic background. **Table S2.** Eating quality traits and agronomic traits of 41 CSSLs in the ‘Koshihikari’ genetic background in 2016. **Table S3.** Eating quality traits and agronomic traits of 41 CSSLs in the ‘Koshihikari’ genetic background in 2017. **Table S4.** Eating quality traits and agronomic traits of 39 CSSLs in the ‘Takanari’ genetic background in 2018. **Table S5.** Eating quality traits and agronomic traits of eight CSSLs carrying chromosome segments from five *indica* rice cultivars in the ‘Koshihikari’ genetic background in 2018.

## Data Availability

The all datasets supporting the conclusions of this article are included in the article and supplementary files.

## Abbreviations

QTL: Quantitative trait locus
CSSL: Chromosome segment substitution line
InDel: Insertion/deletion polymorphism
