# Supplementary material for: Genomic Regions Involved in Differences in Eating and Cooking Quality Other than Wx and Alk Genes between indica and japonica Rice Cultivars
Source: Rice (N Y). 2021 Jan 7;14:8. doi: 10.1186/s12284-020-00447-8 (PMC7790929; doi:10.1186/s12284-020-00447-8)
Supplement: Supplementary file 1 — Additional file 1: Figure S1. Eating quality score, amylose content, protein content, stickiness of the surface of cooked rice grains, hardness of whole cooked rice grains and whiteness of rice grains in all chromosome segment substitution lines in the ‘Koshihikari’ genetic background in 2016 (upper) and 2017 (middle), and in the ‘Takanari’ genetic background in 2018 (lower). Data for eating quality traits are presented as means ± SD (n = 6). [file 12284_2020_447_MOESM1_ESM.pptx]

## Slide 1
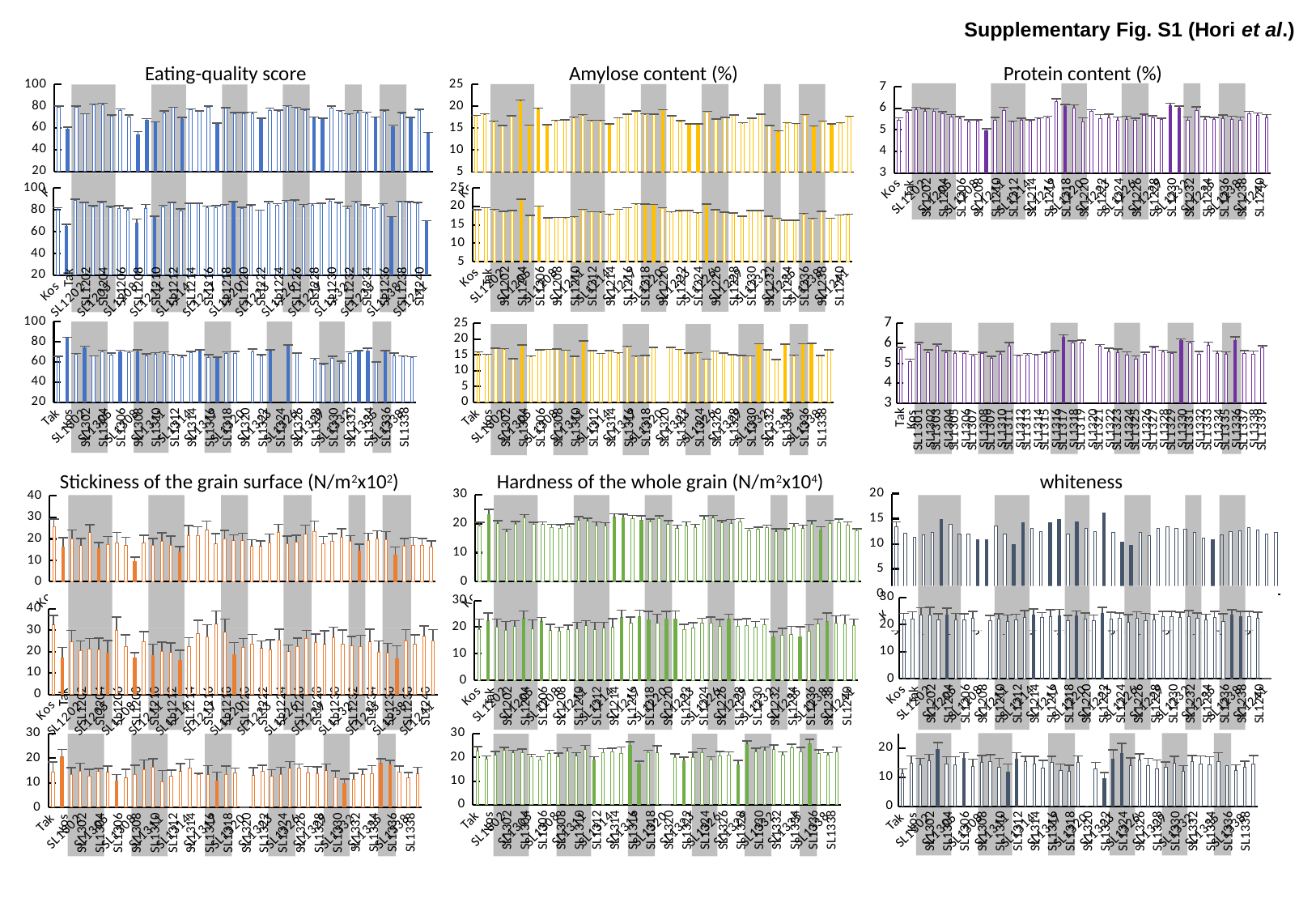

Supplementary Fig. S1 (Hori et al.)
Eating-quality score
Amylose content (%)
Protein content (%)
### Chart
| Category | |
|---|---|
| Kos | 5.4569 |
| Tak | 5.8073 |
| SL1201 | 5.9474 |
| SL1202 | 5.8415 |
| SL1203 | 5.8686 |
| SL1204 | 5.7452 |
| SL1205 | 5.5944 |
| SL1206 | 5.5125 |
| SL1207 | 5.3734 |
| SL1208 | 5.4068 |
| SL1209 | 4.9425 |
| SL1210 | 5.4415 |
| SL1211 | 5.8808 |
| SL1212 | 5.3597 |
| SL1213 | 5.445 |
| SL1214 | 5.4028 |
| SL1215 | 5.5063 |
| SL1216 | 5.5434 |
| SL1217 | 6.2946 |
| SL1218 | 6.0718 |
| SL1219 | 5.9868 |
| SL1220 | 5.358 |
| SL1221 | 5.8369 |
| SL1222 | 5.5193 |
| SL1223 | 5.5618 |
| SL1224 | 5.4593 |
| SL1225 | 5.4784 |
| SL1226 | 5.424 |
| SL1227 | 5.6699 |
| SL1228 | 5.5584 |
| SL1229 | 5.4902 |
| SL1230 | 6.1211 |
| SL1231 | 6.0082 |
| SL1232 | 5.458 |
| SL1233 | 5.8916 |
| SL1234 | 5.4765 |
| SL1235 | 5.4627 |
| SL1236 | 5.5027 |
| SL1237 | 5.4872 |
| SL1238 | 5.434 |
| SL1239 | 5.7519 |
| SL1240 | 5.6756 |
| SL1241 | 5.5585 |
### Chart
| Category | |
|---|---|
| Kos | 79.0 |
| Tak | 59.0 |
| SL1201 | 79.0 |
| SL1202 | 73.0 |
| SL1203 | 81.0 |
| SL1204 | 81.0 |
| SL1205 | 71.0 |
| SL1206 | 76.0 |
| SL1207 | 70.0 |
| SL1208 | 54.0 |
| SL1209 | 67.0 |
| SL1210 | 65.0 |
| SL1211 | 74.0 |
| SL1212 | 79.0 |
| SL1213 | 68.0 |
| SL1214 | 77.0 |
| SL1215 | 75.0 |
| SL1216 | 79.0 |
| SL1217 | 63.0 |
| SL1218 | 78.0 |
| SL1219 | 73.0 |
| SL1220 | 73.0 |
| SL1221 | 73.0 |
| SL1222 | 69.0 |
| SL1223 | 76.0 |
| SL1224 | 75.0 |
| SL1225 | 80.0 |
| SL1226 | 78.0 |
| SL1227 | 76.0 |
| SL1228 | 69.0 |
| SL1229 | 68.0 |
| SL1230 | 78.0 |
| SL1231 | 75.0 |
| SL1232 | 72.0 |
| SL1233 | 74.0 |
| SL1234 | 73.0 |
| SL1235 | 69.0 |
| SL1236 | 75.0 |
| SL1237 | 61.0 |
| SL1238 | 73.0 |
| SL1239 | 69.0 |
| SL1240 | 76.0 |
| SL1241 | 55.0 |
### Chart
| Category | |
|---|---|
| Kos | 17.831494797016656 |
| Tak | 18.155843865838953 |
| SL1201 | 16.534098521727483 |
| SL1202 | 15.51471573400027 |
| SL1203 | 17.78515921575633 |
| SL1204 | 21.306663391541242 |
| SL1205 | 15.607386896520925 |
| SL1206 | 19.453240141128127 |
| SL1207 | 15.70005805904158 |
| SL1208 | 16.76577642802912 |
| SL1209 | 16.81211200928945 |
| SL1210 | 17.414474565673707 |
| SL1211 | 17.924165959537312 |
| SL1212 | 16.76577642802912 |
| SL1213 | 16.71944084676879 |
| SL1214 | 15.978071546603548 |
| SL1215 | 17.36813898441338 |
| SL1216 | 18.063172703318294 |
| SL1217 | 18.758206422223214 |
| SL1218 | 18.155843865838953 |
| SL1219 | 18.063172703318294 |
| SL1220 | 19.03621990978518 |
| SL1221 | 17.738823634496 |
| SL1222 | 16.534098521727483 |
| SL1223 | 15.839064802822564 |
| SL1224 | 15.839064802822564 |
| SL1225 | 18.66553525970256 |
| SL1226 | 16.951118753070432 |
| SL1227 | 17.460810146934037 |
| SL1228 | 17.924165959537312 |
| SL1229 | 16.209749452905186 |
| SL1230 | 17.18279665937207 |
| SL1231 | 18.155843865838953 |
| SL1232 | 15.51471573400027 |
| SL1233 | 14.263655039971416 |
| SL1234 | 16.16341387164486 |
| SL1235 | 15.978071546603548 |
| SL1236 | 17.924165959537312 |
| SL1237 | 15.375708990219286 |
| SL1238 | 16.487762940467157 |
| SL1239 | 15.839064802822564 |
| SL1240 | 16.16341387164486 |
| SL1241 | 17.6494797016658 |
Tak
SL1206
SL1208
SL1210
SL1212
SL1214
SL1216
SL1218
SL1220
SL1222
SL1224
SL1226
SL1228
SL1202
SL1204
SL1230
SL1232
SL1234
SL1236
SL1238
SL1240
### Chart
| Category | |
|---|---|
| Kos | 80.0 |
| Tak | 65.0 |
| SL1201 | 89.0 |
| SL1202 | 87.0 |
| SL1203 | 83.0 |
| SL1204 | 86.33333333333333 |
| SL1205 | 82.0 |
| SL1206 | 81.33333333333333 |
| SL1207 | 80.0 |
| SL1208 | 68.0 |
| SL1209 | 81.66666666666667 |
| SL1210 | 73.66666666666667 |
| SL1211 | 82.66666666666667 |
| SL1212 | 87.0 |
| SL1213 | 79.33333333333333 |
| SL1214 | 85.66666666666667 |
| SL1215 | 85.66666666666667 |
| SL1216 | 82.33333333333333 |
| SL1217 | 82.33333333333333 |
| SL1218 | 84.33333333333333 |
| SL1219 | 86.33333333333333 |
| SL1220 | 81.33333333333333 |
| SL1221 | 83.33333333333333 |
| SL1222 | 80.0 |
| SL1223 | 85.66666666666667 |
| SL1224 | 84.66666666666667 |
| SL1225 | 87.33333333333333 |
| SL1226 | 88.0 |
| SL1227 | 82.66666666666667 |
| SL1228 | 84.66666666666667 |
| SL1229 | 86.0 |
| SL1230 | 87.66666666666667 |
| SL1231 | 86.0 |
| SL1232 | 81.0 |
| SL1233 | 86.66666666666667 |
| SL1234 | 83.33333333333333 |
| SL1235 | 81.0 |
| SL1236 | 84.66666666666667 |
| SL1237 | 73.0 |
| SL1238 | 87.0 |
| SL1239 | 86.33333333333333 |
| SL1240 | 86.0 |
| SL1241 | 70.0 |
### Chart
| Category | |
|---|---|
| Kos | 19.03621990978518 |
| Tak | 19.592246884909112 |
| SL1201 | 19.128891072305837 |
| SL1202 | 18.572864097181903 |
| SL1203 | 18.85087758474387 |
| SL1204 | 21.909025947925507 |
| SL1205 | 17.460810146934037 |
| SL1206 | 20.05560269751239 |
| SL1207 | 16.812112009289446 |
| SL1208 | 16.997454334330758 |
| SL1209 | 16.904783171810102 |
| SL1210 | 17.090125496851414 |
| SL1211 | 19.128891072305837 |
| SL1212 | 18.572864097181903 |
| SL1213 | 18.480192934661247 |
| SL1214 | 17.831494797016656 |
| SL1215 | 19.221562234826493 |
| SL1216 | 19.592246884909112 |
| SL1217 | 20.611629672636326 |
| SL1218 | 20.51895851011567 |
| SL1219 | 20.426287347595014 |
| SL1220 | 19.499575722388457 |
| SL1221 | 18.480192934661247 |
| SL1222 | 18.66553525970256 |
| SL1223 | 18.758206422223214 |
| SL1224 | 18.20217944709928 |
| SL1225 | 20.51895851011567 |
| SL1226 | 19.03621990978518 |
| SL1227 | 18.38752177214059 |
| SL1228 | 18.20217944709928 |
| SL1229 | 17.36813898441338 |
| SL1230 | 18.758206422223214 |
| SL1231 | 18.85087758474387 |
| SL1232 | 17.18279665937207 |
| SL1233 | 16.62676968424814 |
| SL1234 | 16.070742709124204 |
| SL1235 | 16.16341387164486 |
| SL1236 | 17.924165959537312 |
| SL1237 | 16.62676968424814 |
| SL1238 | 18.480192934661247 |
| SL1239 | 16.719440846768794 |
| SL1240 | 17.646152471975345 |
| SL1241 | 17.738823634496 |Tak
Tak
SL1206
SL1208
SL1210
SL1212
SL1214
SL1216
SL1218
SL1220
SL1222
SL1224
SL1226
SL1228
SL1202
SL1204
SL1230
SL1232
SL1234
SL1236
SL1238
SL1240
SL1202
SL1204
SL1206
SL1208
SL1210
SL1212
SL1214
SL1216
SL1218
SL1220
SL1222
SL1224
SL1226
SL1228
SL1230
SL1232
SL1234
SL1236
SL1238
SL1240
### Chart
| Category | |
|---|---|
| Tak | 64.0 |
| Kos | 84.33333333333333 |
| SL1301 | 67.33333333333333 |
| SL1302 | 74.0 |
| SL1303 | 66.0 |
| SL1304 | 69.66666666666667 |
| SL1305 | 67.0 |
| SL1306 | 70.0 |
| SL1307 | 69.0 |
| SL1308 | 70.0 |
| SL1309 | 66.33333333333333 |
| SL1310 | 67.66666666666667 |
| SL1311 | 68.66666666666667 |
| SL1312 | 65.66666666666667 |
| SL1313 | 64.66666666666667 |
| SL1314 | 69.33333333333333 |
| SL1315 | 70.66666666666667 |
| SL1316 | 64.66666666666667 |
| SL1317 | 63.333333333333336 |
| SL1318 | 68.33333333333333 |
| SL1319 | 68.33333333333333 |
| SL1320 | 0.0 |
| SL1321 | 69.66666666666667 |
| SL1322 | 65.66666666666667 |
| SL1323 | 70.66666666666667 |
| SL1324 | 0.0 |
| SL1325 | 76.0 |
| SL1326 | 68.33333333333333 |
| SL1327 | 0.0 |
| SL1328 | 62.0 |
| SL1329 | 57.333333333333336 |
| SL1330 | 63.333333333333336 |
| SL1331 | 58.666666666666664 |
| SL1332 | 68.0 |
| SL1333 | 71.0 |
| SL1334 | 71.0 |
| SL1335 | 60.0 |
| SL1336 | 71.0 |
| SL1337 | 66.33333333333333 |
| SL1338 | 65.33333333333333 |
| SL1339 | 64.66666666666667 |
### Chart
| Category | |
|---|---|
| Tak | 15.8142284569138 |
| Kos | 15.060692464358452 |
| SL1301 | 16.967058823529413 |
| SL1302 | 16.80697167755991 |
| SL1303 | 13.6989106753813 |
| SL1304 | 18.004452690166975 |
| SL1305 | 14.518609406952963 |
| SL1306 | 16.467583497053045 |
| SL1307 | 16.685017421602787 |
| SL1308 | 16.831638418079095 |
| SL1309 | 16.421487603305785 |
| SL1310 | 14.331107205623901 |
| SL1311 | 19.19853211009174 |
| SL1312 | 16.27615894039735 |
| SL1313 | 15.357221206581352 |
| SL1314 | 16.34750462107209 |
| SL1315 | 15.622710622710622 |
| SL1316 | 17.63014705882353 |
| SL1317 | 14.500757575757575 |
| SL1318 | 14.702762430939226 |
| SL1319 | 17.206142034548943 |
| SL1320 | 0.0 |
| SL1321 | 17.31159420289855 |
| SL1322 | 16.5556420233463 |
| SL1323 | 15.470707070707071 |
| SL1324 | 15.5513126491647 |
| SL1325 | 13.523694779116465 |
| SL1326 | 16.12041237113402 |
| SL1327 | 15.466666666666665 |
| SL1328 | 15.017118997912318 |
| SL1329 | 14.7550200803213 |
| SL1330 | 14.574517374517374 |
| SL1331 | 18.4335238095238 |
| SL1332 | 16.42201138519924 |
| SL1333 | 13.3540308747855 |
| SL1334 | 18.230842607313196 |
| SL1335 | 14.8102040816327 |
| SL1336 | 18.3436325678497 |
| SL1337 | 18.5544 |
| SL1338 | 14.64070796460177 |
| SL1339 | 16.4842911877395 |
### Chart
| Category | |
|---|---|
| Tak | 5.683866666666667 |
| Kos | 5.104166666666667 |
| SL1301 | 5.943466666666667 |
| SL1302 | 5.538966666666667 |
| SL1303 | 5.872666666666667 |
| SL1304 | 5.544733333333333 |
| SL1305 | 5.505966666666667 |
| SL1306 | 5.507066666666667 |
| SL1307 | 5.352233333333333 |
| SL1308 | 5.494466666666667 |
| SL1309 | 5.243366666666667 |
| SL1310 | 5.446933333333333 |
| SL1311 | 5.8671999999999995 |
| SL1312 | 5.352833333333333 |
| SL1313 | 5.407 |
| SL1314 | 5.404966666666667 |
| SL1315 | 5.503033333333334 |
| SL1316 | 5.5427 |
| SL1317 | 6.283333333333334 |
| SL1318 | 6.0185 |
| SL1319 | 6.0004 |
| SL1320 | 0.0 |
| SL1321 | 5.842666666666666 |
| SL1322 | 5.5529 |
| SL1323 | 5.545466666666667 |
| SL1324 | 5.427266666666665 |
| SL1325 | 5.214533333333333 |
| SL1326 | 5.441 |
| SL1327 | 5.7864 |
| SL1328 | 5.552466666666667 |
| SL1329 | 5.4949 |
| SL1330 | 6.121433333333333 |
| SL1331 | 6.003166666666666 |
| SL1332 | 5.456333333333333 |
| SL1333 | 5.897366666666667 |
| SL1334 | 5.479900000000001 |
| SL1335 | 5.460999999999999 |
| SL1336 | 6.1462 |
| SL1337 | 5.493266666666667 |
| SL1338 | 5.4436 |
| SL1339 | 5.761433333333334 |
Kos
SL1332
SL1334
SL1336
SL1338
SL1314
SL1316
SL1318
SL1320
SL1322
SL1324
SL1302
SL1304
SL1306
SL1308
SL1310
SL1312
SL1326
SL1328
SL1330
Kos
Kos
SL1332
SL1334
SL1336
SL1338
SL1332
SL1334
SL1336
SL1338
SL1314
SL1316
SL1318
SL1320
SL1322
SL1324
SL1302
SL1304
SL1306
SL1308
SL1310
SL1312
SL1326
SL1328
SL1330
SL1314
SL1316
SL1318
SL1320
SL1322
SL1324
SL1302
SL1304
SL1306
SL1308
SL1310
SL1312
SL1326
SL1328
SL1330
Stickiness of the grain surface (N/m2x102)
Hardness of the whole grain (N/m2x104)
whiteness
### Chart
| Category | |
|---|---|
| Kos | 25.81 |
| Tak | 16.34 |
| SL1201 | 20.04 |
| SL1202 | 16.96 |
| SL1203 | 22.86 |
| SL1204 | 15.56 |
| SL1205 | 17.4 |
| SL1206 | 18.18 |
| SL1207 | 16.97 |
| SL1208 | 9.454 |
| SL1209 | 18.04 |
| SL1210 | 17.1 |
| SL1211 | 19.01 |
| SL1212 | 17.04 |
| SL1213 | 14.02 |
| SL1214 | 21.6 |
| SL1215 | 21.53 |
| SL1216 | 24.1 |
| SL1217 | 17.67 |
| SL1218 | 20.06 |
| SL1219 | 19.15 |
| SL1220 | 19.06 |
| SL1221 | 16.52 |
| SL1222 | 16.51 |
| SL1223 | 18.22 |
| SL1224 | 23.06 |
| SL1225 | 17.88 |
| SL1226 | 18.5 |
| SL1227 | 22.16 |
| SL1228 | 23.54 |
| SL1229 | 17.55 |
| SL1230 | 18.74 |
| SL1231 | 20.83 |
| SL1232 | 18.91 |
| SL1233 | 14.36 |
| SL1234 | 19.34 |
| SL1235 | 20.04 |
| SL1236 | 19.74 |
| SL1237 | 12.58 |
| SL1238 | 16.74 |
| SL1239 | 16.99 |
| SL1240 | 17.04 |
| SL1241 | 16.34 |
### Chart
| Category | |
|---|---|
| Kos | 19.3 |
| Tak | 23.07 |
| SL1201 | 19.99 |
| SL1202 | 17.1 |
| SL1203 | 19.77 |
| SL1204 | 21.96 |
| SL1205 | 19.63 |
| SL1206 | 19.7 |
| SL1207 | 18.73 |
| SL1208 | 18.39 |
| SL1209 | 18.92 |
| SL1210 | 21.01 |
| SL1211 | 20.76 |
| SL1212 | 19.29 |
| SL1213 | 19.17 |
| SL1214 | 22.23 |
| SL1215 | 22.05 |
| SL1216 | 21.67 |
| SL1217 | 21.11 |
| SL1218 | 20.65 |
| SL1219 | 21.7 |
| SL1220 | 19.66 |
| SL1221 | 18.34 |
| SL1222 | 19.24 |
| SL1223 | 18.71 |
| SL1224 | 21.52 |
| SL1225 | 21.82 |
| SL1226 | 20.2 |
| SL1227 | 20.0 |
| SL1228 | 20.65 |
| SL1229 | 17.61 |
| SL1230 | 18.01 |
| SL1231 | 18.59 |
| SL1232 | 17.17 |
| SL1233 | 17.58 |
| SL1234 | 19.02 |
| SL1235 | 18.34 |
| SL1236 | 19.81 |
| SL1237 | 17.91 |
| SL1238 | 19.94 |
| SL1239 | 20.43 |
| SL1240 | 19.43 |
| SL1241 | 17.62 |
### Chart
| Category | |
|---|---|
| Kos | 13.36 |
| Tak | 12.02 |
| SL1201 | 11.35 |
| SL1202 | 11.77 |
| SL1203 | 12.33 |
| SL1204 | 14.87 |
| SL1205 | 13.93 |
| SL1206 | 11.87 |
| SL1207 | 11.91 |
| SL1208 | 10.78 |
| SL1209 | 10.8 |
| SL1210 | 13.56 |
| SL1211 | 11.89 |
| SL1212 | 9.88 |
| SL1213 | 14.25 |
| SL1214 | 13.07 |
| SL1215 | 12.46 |
| SL1216 | 14.25 |
| SL1217 | 14.83 |
| SL1218 | 11.97 |
| SL1219 | 14.38 |
| SL1220 | 13.08 |
| SL1221 | 12.38 |
| SL1222 | 16.21 |
| SL1223 | 12.31 |
| SL1224 | 10.37 |
| SL1225 | 9.64 |
| SL1226 | 12.29 |
| SL1227 | 11.66 |
| SL1228 | 13.0 |
| SL1229 | 13.45 |
| SL1230 | 13.12 |
| SL1231 | 12.84 |
| SL1232 | 12.21 |
| SL1233 | 11.11 |
| SL1234 | 10.74 |
| SL1235 | 11.76 |
| SL1236 | 12.44 |
| SL1237 | 12.59 |
| SL1238 | 13.28 |
| SL1239 | 12.68 |
| SL1240 | 11.95 |
| SL1241 | 12.3 |
### Chart
| Category | |
|---|---|
| Kos | 21.6803 |
| Tak | 21.8492 |
| SL1201 | 23.4032 |
| SL1202 | 23.3606 |
| SL1203 | 21.834 |
| SL1204 | 23.4884 |
| SL1205 | 21.6835 |
| SL1206 | 21.848 |
| SL1207 | 22.4247 |
| SL1208 | 0.0 |
| SL1209 | 21.291 |
| SL1210 | 21.8587 |
| SL1211 | 21.0488 |
| SL1212 | 21.592 |
| SL1213 | 22.5229 |
| SL1214 | 23.5854 |
| SL1215 | 22.4532 |
| SL1216 | 22.8596 |
| SL1217 | 23.2412 |
| SL1218 | 21.2777 |
| SL1219 | 23.1377 |
| SL1220 | 22.0327 |
| SL1221 | 21.4516 |
| SL1222 | 24.1938 |
| SL1223 | 22.023 |
| SL1224 | 22.1554 |
| SL1225 | 20.7385 |
| SL1226 | 22.4102 |
| SL1227 | 21.5164 |
| SL1228 | 21.8011 |
| SL1229 | 22.8597 |
| SL1230 | 22.7426 |
| SL1231 | 22.5396 |
| SL1232 | 22.987 |
| SL1233 | 22.3503 |
| SL1234 | 21.6206 |
| SL1235 | 22.6432 |
| SL1236 | 21.2 |
| SL1237 | 23.3491 |
| SL1238 | 23.0107 |
| SL1239 | 22.7857 |
| SL1240 | 22.2382 |
| SL1241 | 0.0 |
### Chart
| Category | |
|---|---|
| Kos | 19.19 |
| Tak | 22.33 |
| SL1201 | 20.07 |
| SL1202 | 18.86 |
| SL1203 | 20.17 |
| SL1204 | 23.25 |
| SL1205 | 19.34 |
| SL1206 | 22.04 |
| SL1207 | 18.66 |
| SL1208 | 18.54 |
| SL1209 | 19.04 |
| SL1210 | 19.35 |
| SL1211 | 20.51 |
| SL1212 | 18.96 |
| SL1213 | 19.61 |
| SL1214 | 19.95 |
| SL1215 | 23.56 |
| SL1216 | 21.37 |
| SL1217 | 23.92 |
| SL1218 | 22.81 |
| SL1219 | 21.41 |
| SL1220 | 23.07 |
| SL1221 | 23.07 |
| SL1222 | 19.14 |
| SL1223 | 19.63 |
| SL1224 | 21.49 |
| SL1225 | 21.51 |
| SL1226 | 20.27 |
| SL1227 | 22.97 |
| SL1228 | 20.39 |
| SL1229 | 20.68 |
| SL1230 | 19.87 |
| SL1231 | 20.79 |
| SL1232 | 16.3 |
| SL1233 | 17.09 |
| SL1234 | 17.11 |
| SL1235 | 16.39 |
| SL1236 | 18.32 |
| SL1237 | 21.14 |
| SL1238 | 21.98 |
| SL1239 | 21.37 |
| SL1240 | 21.33 |
| SL1241 | 20.62 |
### Chart
| Category | |
|---|---|
| Kos | 32.35 |
| Tak | 17.19 |
| SL1201 | 24.38 |
| SL1202 | 20.47 |
| SL1203 | 21.24 |
| SL1204 | 20.94 |
| SL1205 | 19.37 |
| SL1206 | 30.02 |
| SL1207 | 22.39 |
| SL1208 | 17.1 |
| SL1209 | 24.74 |
| SL1210 | 18.32 |
| SL1211 | 20.17 |
| SL1212 | 19.63 |
| SL1213 | 15.88 |
| SL1214 | 22.29 |
| SL1215 | 28.24 |
| SL1216 | 26.9 |
| SL1217 | 32.84 |
| SL1218 | 29.0 |
| SL1219 | 18.63 |
| SL1220 | 22.11 |
| SL1221 | 23.47 |
| SL1222 | 21.48 |
| SL1223 | 20.96 |
| SL1224 | 25.45 |
| SL1225 | 19.92 |
| SL1226 | 22.4 |
| SL1227 | 26.18 |
| SL1228 | 24.32 |
| SL1229 | 23.38 |
| SL1230 | 26.56 |
| SL1231 | 23.52 |
| SL1232 | 22.6 |
| SL1233 | 22.41 |
| SL1234 | 24.42 |
| SL1235 | 19.83 |
| SL1236 | 19.37 |
| SL1237 | 16.82 |
| SL1238 | 25.12 |
| SL1239 | 23.42 |
| SL1240 | 27.12 |
| SL1241 | 25.06 |Tak
Tak
Tak
SL1224
SL1226
SL1228
SL1230
SL1232
SL1234
SL1236
SL1238
SL1240
SL1202
SL1204
SL1206
SL1208
SL1210
SL1212
SL1214
SL1216
SL1218
SL1220
SL1222
SL1202
SL1204
SL1206
SL1208
SL1210
SL1212
SL1214
SL1216
SL1218
SL1220
SL1222
SL1224
SL1226
SL1228
SL1230
SL1232
SL1234
SL1236
SL1238
SL1240
SL1202
SL1204
SL1206
SL1208
SL1210
SL1212
SL1214
SL1216
SL1218
SL1220
SL1222
SL1224
SL1226
SL1228
SL1230
SL1232
SL1234
SL1236
SL1238
SL1240
### Chart
| Category | |
|---|---|
| Tak | 14.44 |
| Kos | 20.49 |
| SL1301 | 13.49 |
| SL1302 | 14.74 |
| SL1303 | 12.78 |
| SL1304 | 14.58 |
| SL1305 | 14.28 |
| SL1306 | 10.84 |
| SL1307 | 12.03 |
| SL1308 | 13.45 |
| SL1309 | 15.45 |
| SL1310 | 16.49 |
| SL1311 | 10.48 |
| SL1312 | 12.64 |
| SL1313 | 14.68 |
| SL1314 | 15.96 |
| SL1315 | 13.3 |
| SL1316 | 13.33 |
| SL1317 | 10.73 |
| SL1318 | 13.41 |
| SL1319 | 14.0 |
| SL1320 | 0.0 |
| SL1321 | 12.93 |
| SL1322 | 14.53 |
| SL1323 | 12.58 |
| SL1324 | 13.42 |
| SL1325 | 15.87 |
| SL1326 | 15.85 |
| SL1327 | 14.11 |
| SL1328 | 13.73 |
| SL1329 | 14.88 |
| SL1330 | 12.07 |
| SL1331 | 9.751 |
| SL1332 | 11.53 |
| SL1333 | 13.31 |
| SL1334 | 13.7 |
| SL1335 | 17.82 |
| SL1336 | 17.39 |
| SL1337 | 14.25 |
| SL1338 | 11.9 |
| SL1339 | 13.64 |
### Chart
| Category | |
|---|---|
| Tak | 11.18 |
| Kos | 14.74 |
| SL1301 | 14.27 |
| SL1302 | 15.68 |
| SL1303 | 19.53 |
| SL1304 | 14.54 |
| SL1305 | 14.26 |
| SL1306 | 16.34 |
| SL1307 | 13.61 |
| SL1308 | 14.99 |
| SL1309 | 15.47 |
| SL1310 | 13.51 |
| SL1311 | 11.8 |
| SL1312 | 16.18 |
| SL1313 | 15.28 |
| SL1314 | 14.58 |
| SL1315 | 13.23 |
| SL1316 | 15.17 |
| SL1317 | 12.3 |
| SL1318 | 12.09 |
| SL1319 | 15.1 |
| SL1320 | 0.0 |
| SL1321 | 12.75 |
| SL1322 | 9.44 |
| SL1323 | 16.18 |
| SL1324 | 18.16 |
| SL1325 | 13.88 |
| SL1326 | 15.92 |
| SL1327 | 14.03 |
| SL1328 | 12.75 |
| SL1329 | 13.31 |
| SL1330 | 14.88 |
| SL1331 | 11.99 |
| SL1332 | 15.34 |
| SL1333 | 14.48 |
| SL1334 | 14.33 |
| SL1335 | 15.32 |
| SL1336 | 13.9 |
| SL1337 | 12.41 |
| SL1338 | 13.35 |
| SL1339 | 14.49 |
### Chart
| Category | |
|---|---|
| Tak | 22.8 |
| Kos | 19.37 |
| SL1301 | 20.92 |
| SL1302 | 22.97 |
| SL1303 | 21.95 |
| SL1304 | 21.82 |
| SL1305 | 20.18 |
| SL1306 | 19.04 |
| SL1307 | 21.65 |
| SL1308 | 20.22 |
| SL1309 | 22.61 |
| SL1310 | 20.49 |
| SL1311 | 23.34 |
| SL1312 | 18.95 |
| SL1313 | 21.98 |
| SL1314 | 22.36 |
| SL1315 | 21.67 |
| SL1316 | 25.05 |
| SL1317 | 17.35 |
| SL1318 | 22.08 |
| SL1319 | 21.84 |
| SL1320 | 0.0 |
| SL1321 | 20.08 |
| SL1322 | 18.7 |
| SL1323 | 19.92 |
| SL1324 | 22.02 |
| SL1325 | 19.02 |
| SL1326 | 20.71 |
| SL1327 | 20.95 |
| SL1328 | 17.01 |
| SL1329 | 25.53 |
| SL1330 | 22.32 |
| SL1331 | 22.85 |
| SL1332 | 23.26 |
| SL1333 | 20.98 |
| SL1334 | 23.91 |
| SL1335 | 22.33 |
| SL1336 | 25.58 |
| SL1337 | 21.48 |
| SL1338 | 21.06 |
| SL1339 | 22.45 |
Kos
Kos
Kos
SL1332
SL1334
SL1336
SL1338
SL1314
SL1316
SL1318
SL1320
SL1322
SL1324
SL1302
SL1304
SL1306
SL1308
SL1310
SL1312
SL1326
SL1328
SL1330
SL1324
SL1326
SL1328
SL1330
SL1332
SL1334
SL1336
SL1338
SL1302
SL1304
SL1306
SL1308
SL1310
SL1312
SL1314
SL1316
SL1318
SL1320
SL1322
SL1332
SL1334
SL1336
SL1338
SL1314
SL1316
SL1318
SL1320
SL1322
SL1324
SL1302
SL1304
SL1306
SL1308
SL1310
SL1312
SL1326
SL1328
SL1330
